# Plant-Derived Toxin Inhibitors as Potential Candidates to Complement Antivenom Treatment in Snakebite Envenomations

**DOI:** 10.3389/fimmu.2022.842576

**Published:** 2022-05-09

**Authors:** Asenate A. X. Adrião, Aline O. dos Santos, Emilly J. S. P. de Lima, Jéssica B. Maciel, Weider H. P. Paz, Felipe M. A. da Silva, Manuela B. Pucca, Ana M. Moura-da-Silva, Wuelton M. Monteiro, Marco A. Sartim, Hector H. F. Koolen

**Affiliations:** ^1^Post Graduate Program in Biodiversity and Biotechnology BIONORTE, Superior School of Health Sciences, Amazonas State University, Manaus, Brazil; ^2^Post Graduate Program in Tropical Medicine, Department of Teaching and Research, Dr. Heitor Vieira Dourado Tropical Medicine Foundation, Manaus, Brazil; ^3^Post Graduate Program in Chemistry, Department of Chemistry, Federal University of Amazonas, Manaus, Brazil; ^4^Multidisciplinary Support Center, Federal University of Amazonas, Manaus, Brazil; ^5^Medical School, Federal University of Roraima, Boa Vista, Brazil; ^6^Laboratory of Immunopathology, Institute Butantan, São Paulo, Brazil; ^7^University Nilton Lins, Manaus, Brazil

**Keywords:** bioactive compounds, plants, envenomation, snakes, snakebites

## Abstract

Snakebite envenomations (SBEs) are a neglected medical condition of global importance that mainly affect the tropical and subtropical regions. Clinical manifestations include pain, edema, hemorrhage, tissue necrosis, and neurotoxic signs, and may evolve to functional loss of the affected limb, acute renal and/or respiratory failure, and even death. The standard treatment for snake envenomations is antivenom, which is produced from the hyperimmunization of animals with snake toxins. The inhibition of the effects of SBEs using natural or synthetic compounds has been suggested as a complementary treatment particularly before admission to hospital for antivenom treatment, since these alternative molecules are also able to inhibit toxins. Biodiversity-derived molecules, namely those extracted from medicinal plants, are promising sources of toxin inhibitors that can minimize the deleterious consequences of SBEs. In this review, we systematically synthesize the literature on plant metabolites that can be used as toxin-inhibiting agents, as well as present the potential mechanisms of action of molecules derived from natural sources. These findings aim to further our understanding of the potential of natural products and provide new lead compounds as auxiliary therapies for SBEs.

## Snakebite Envenomings

Snakebite envenomations (SBEs) represent a serious and neglected public health problem that occurs worldwide, especially in developing countries in tropical and subtropical regions (1, 2). These countries have high incidences of cases because, to some extent, they still conserve their forests and biodiversity; however, at the same time, human expansion and urbanization tend to invade places where biodiversity is greater and this leads to an increase in contact between humans and snakes (2, 3), especially in the countries of Asia, Sub-Saharan Africa and Latin America (4).

**Figure 1:**
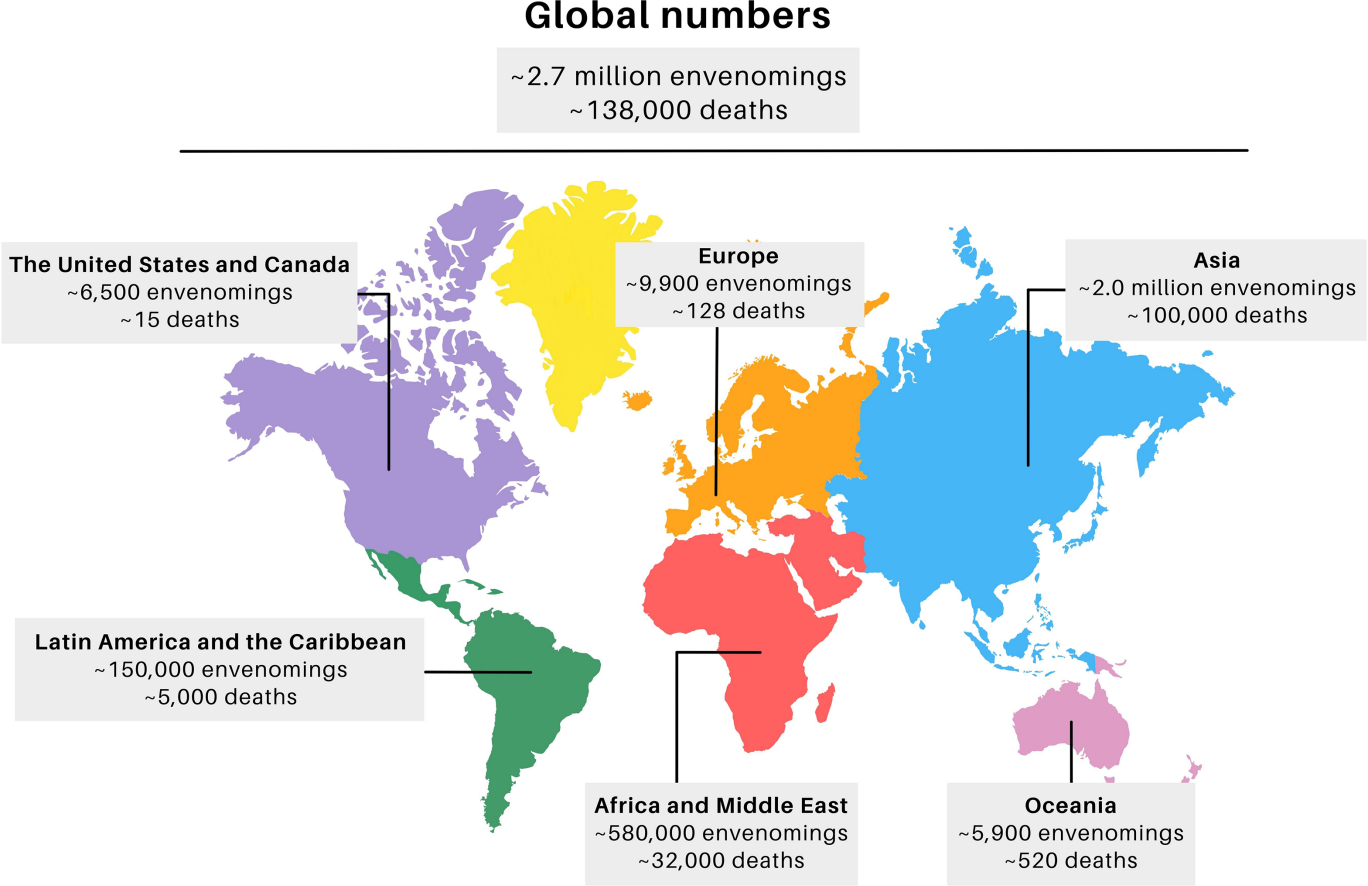
Global distribution of snakebite cases. Adapted from J.M. Gutiérrez et al. (2017) Ref. (6) The final figure was prepared using canva.com.

Worldwide, about 1.8 to 2.7 million snakebites are estimated to occur annually, which resulted in about 138,000 deaths and approximately 400,000 cases of people who have permanent physical sequelae (**Figure 1**) (1, 5, 6). The highest incidence occurs in Asia, which presents 73% of the total world cases (~2 million cases), most of them in India, where more than 46,000 deaths were reported in 2020 (1). Africa and the Middle East are in second place, and present about 580,000 SBEs (21%), of which 7,000 to 32,000 deaths occurred in sub-Saharan Africa alone (1, 7, 8). Together Latin America and the Caribbean present about 150,000 SBEs (5%), with 5,000 deaths, most of them in South America with 50,000 cases, particularly in Brazil with 26,000-29,000 cases per year, of which one third occur in the Amazon region (6, 9). These estimates may show lower numbers than what occurs in reality, since a considerable portion of cases go unreported (4). Underreporting occurs due to SBEs occurring in remote rural areas where there is difficulty accessing health services (4, 10).

Among the snakes of greater clinical importance that cause high levels of morbidity or mortality, those that belong to the families Elapidae and Viperidae stand out (11). On the Asian continent, the clinically important species of the family Viperidae, include the genera *Daboia* (e.g., *D. russelii*) and *Echis* (e.g., *E. carinatus* and *E. sochureki*), which inhabit open and dry environments. Other species that cause severe envenomations are the desert vipers, comprising *Macrovipera*, *Eristicophis* and *Pseudocerastes* genera (12). In Asia, snakes belonging to the family Elapidae include *Naja* (e.g., *N. naja*, *N. kaouthia* and *N. oxiana*) and *Bungarus caeruleus* (6, 13).

In regard to the clinically important African species, some of the same Asian genera of the family Elapidae are also reported, such as *Naja* (e.g., *N. haje*, *N. melanoleuca, N. nigricollis* and *N. anchietae*), as well as species of Viperidae from *Bitis* and *Dendroaspis* genera (6, 14). While, in Central and South America, cases predominate with species belonging to the Viperidae, especially *Bothrops* (e.g., *B. atrox*, *B. asper*, *B. jararaca*, *B. alternatus*, *B. jararacussu* and *B. erythromelas*), *Crotalus* (e.g., *C. durissus* and *C. simus*) and *Lachesis* (e.g., *L. muta*), which inhabit the dense forests of this region (15–17).

**Figure 2:**
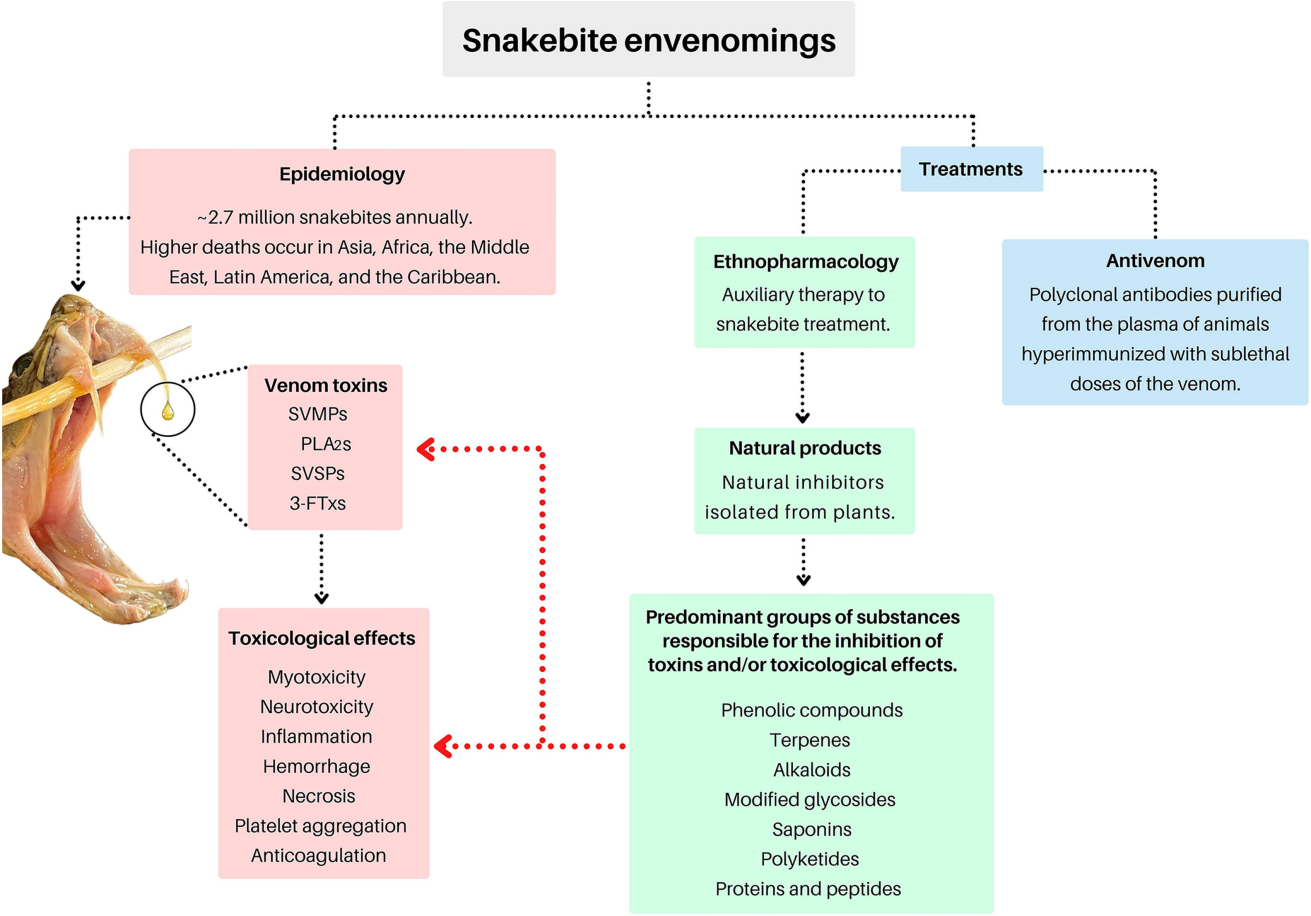
Mind map of the topics covered in this review. Snake photo: Asenate A. X. Adrião. The final figure was prepared using canva.com.

Despite the great advances in health services, the treatment of snakebites is often still a challenge. Although antivenom therapy reduces mortality, it is ineffective against local tissue damage. In addition to these factors, serum availability is low in many distant regions (2). Due to difficulties in accessing treatment, many people have developed their own methods to minimize the damage caused by snakebites (9, 10). It is known that medicinal plants used by traditional healers against snake bites are found all over the world, so the use of extracts, teas from leaves, roots and stem bark of plants is common in many of these countries (9, 10). However, many cases of snakebite envenomation have negative clinical outcomes before the patient receives appropriate treatment, due to the dangerous and unscientific use of substances that can do more harm than good and end up impairing the patient’s treatment by a professional (9, 10). However, exploring ethnobotanical knowledge in order to discover natural inhibitors of snake toxins may also provide new therapeutic treatments in the future. The literature reports many *in vitro* and *in vivo* studies that have demonstrated that bioactive molecules isolated and derived from natural products show antivenom activities (10). Therefore, based on the available literature, this updated review highlights some of the natural bioactive compounds that have been isolated from plants, and may be used as potential adjuvant inhibitors of snake venom toxins, as well as presenting new perspectives regarding their potential use in the development of new therapies for snakebites. **Figure 2** represents an overview of the roadmap proposed in this review.

An extensive literature review was carried out using different scientific electronic sources, including databases such as Scifinder, Pubmed, Scopus, Web of Science and Google Scholar. The study databases included original papers published in peer-reviewed journals, books, dissertations, theses and patents, and all data of scientific information written or translated into English published until November 2021 was considered. The keywords “snakebites, snake envenomation, snake venom, natural inhibitors, antivenom activity, toxins, plants, phospholipase inhibitors and metalloprotease inhibitors” were used individually, but mostly in combination. Data showing the bioactivity of compounds isolated from plants used in *in vitro* and *in vivo* tests against snake venoms, their toxins and/or the biological activities caused by them were considered.

## Venom Toxins

Venoms are used by more than 250,000 species to subdue prey, confuse competitors or in defense against their predators. The evolutionary success of venoms has been evidenced by venomous animals occupying all ecosystems (18). Venomous animals, such as snakes, have their envenomations defined as an injection into the tissue of another animal using specialized teeth, commonly called fangs, and use a glandular secretion rich in toxins to immobilize and digest their food, though envenomation can also be used as a defense and survival tool (19, 20). The proteome of the ancestral venom has diversified among a variety of snake families due to factors such as genetic mutations and natural selection in order to shape and differentiate venoms, thus conferring specific toxicity to each species (21).

**Figure 3:**
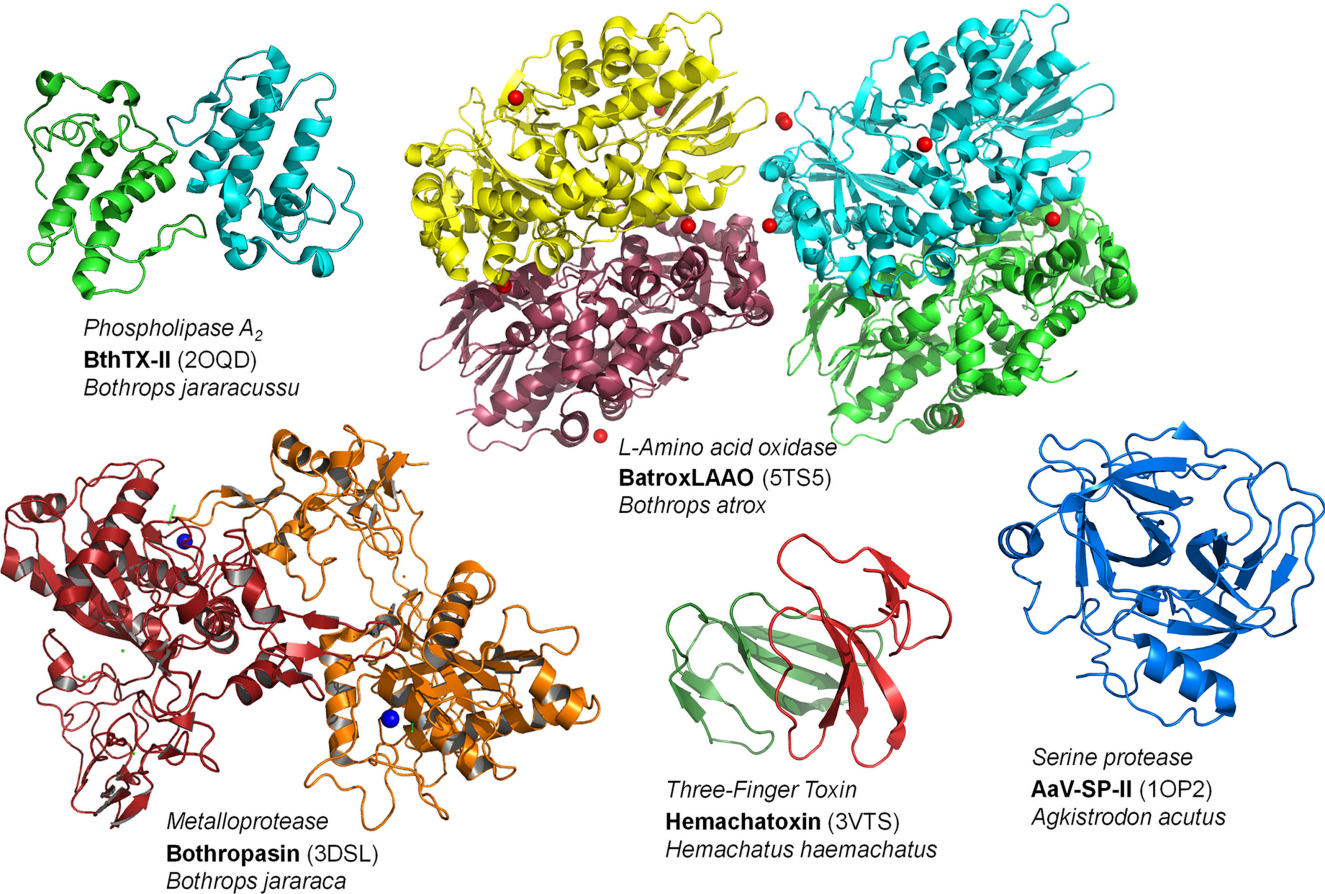
Structures of representatives of the main toxin classes in snake venoms. Codes inside parentheses denote the PDB codes of the crustal strucutures of these proteins. The final figure was prepared using Pymol v. 1.6.

Approximately 90-95% of the dry weight of snake venom corresponds to proteins and peptides that act as toxins, and it may or may not have enzymatic action. This composition can be made up of phospholipases A_2_ (PLA_2_s), metalloproteases (SVMPs), serine proteases (SVSPs), L-amino acid oxidases (LAAOs), phosphodiesterases (PDEs), hyaluronidases (HAases), acetylcolinesterases (AchEs), nucleases, three-finger toxins (3-FTxs), desintegrins, cysteine-rich secretory proteins, and C-type lectins (CTLs) (**Figure 3**) (22). Not all peptides and enzymes are present in all venoms; the synthesis and secretion of the different classes of proteins end up not being synchronized and can thus result in variations in the composition of the venom according to the different stages of the production cycle (23, 24). Although the venom of snakes has more than 20 families of proteins, the most relevant components are found (for the most part) in four of them in varying proportions, thus representing the main targets to be inhibited by natural molecules (25). These proteins are PLA_2_s, SVMPs, SVSPs and 3-FTxs, and these families interact to attack several different physiological targets, which causes the various pathologies described above (26, 27).

It is the action of these toxins within the venoms of different snake species that determine the pathophysiological outcomes in patients. In general, with a few exceptions, venoms of Elapidae snakes are rich sources of neurotoxic PLA_2_s and 3-FTxs, which result in neurotoxic activity. These venoms induce minor local effects, but envenomings may progress to neurotoxicity and acute respiratory failure, a potential life-threatening manifestation (6). On the other hand, viper venoms contain many proteases that cause local and systemic manifestations mainly related to local proteolytic effects and hemostatic disorders (6, 28). Viper venom proteases and PLA_2_s are also involved in tissue damage with local signs and symptoms such as pain, swelling, blistering, bleeding and ecchymosis. The victims may also present complications such as secondary bacterial infection, compartmental syndrome, and necrosis with consequent tissue loss, which leads to amputation in more severe cases. Other systemic complications such as acute kidney injury are often reported (29). One important consideration is that the composition and concentration of each toxin in the venom may vary according to the age, sex, dietary habits, and geographic distribution of the snake (30, 31).

### Phospholipases A2 (PLA_2_s)

PLA_2_s enzymes are commonly found in many taxa, which include bacteria, plants, invertebrates such as arachnids and insects, and in vertebrates, in mammalian tissues and in snake venoms. PLA_2_s belong to subgroup II of secreted phospholipases (PLA_2_s), which is divided into two classes: those that have aspartate amino acid residues at position 49 (Asp49), which can catalyze a hydrolysis of the *sn*-2 acyl bond in phospholipids present in several cells and free lipids; and those that have a lysine residue at position 49 (Lys49), which are catalytically inactive, however, with prominent myotoxic action (32, 33). The PLA_2_s of snake venom have a wide variety of impressive biological effects that act alone or together, and among the effects are neurotoxicity, myotoxicity, cardiotoxicity, anticoagulation, spasms, inhibition of platelet aggregation, hypotension and inflammation (24, 34). Although devoid of catalytic activity, Lys49 PLA_2_s are strongly myotoxic and contribute to the venom-induced tissue-damage. Some examples of isolated PLA_2_s are the toxins BaPLA_2_I and BaPLA_2_III, which belong to the group of PLA_2_s that were isolated from the venom of the snake *B. atrox* (35).

### Snake Venom Metalloproteases (SVMPs)

The SVMPs are enzymes with variable molecular mass (20 to 100 kDa) and are dependent on divalent metallic ions (such as zinc), which are responsible for maintaining their three-dimensional structure to perform their catalytic functions. Its catalytic site is highly conserved among SVMPs and presents the zinc-binding domain HEXXHXXGXXH and the methionine-turn motif. SVMPs are classified in groups and subgroups based on their domain organization. P-I contains only the metalloproteinases domain, P-II contains the metalloproteinases domain followed by disintegrin and P-III presents the metalloproteinases, disintegrin-like and cysteine-rich domains, and a possible additional lectin-like domain, which is classified as PIIId (36). Due to their size, these enzymes can act predominantly in the circulatory system to facilitate their dispersion and the amplification of the toxicity of the components of the venom that have lower molecular weight (37).

SVMPs act as the main factors responsible for envenomation mechanisms, such as local and systemic hemorrhage, necrosis, blisters and further inflammation. These effects are related to their proteolytic action by the protease domain, and associated domains such as the disintegrin/disintegrin-like and lectin-like domains. The hemorrhagic activity results from the cleavage and degrading of structurally important components of the basal membranes (laminin, nidogen, fibronectin, proteoglycans and type IV collagen), which promotes the rupture of connective tissue components that are responsible for the structural integrity of the blood vessels, thus producing local and systemic hemorrhages (38). The loosening of the connective tissue is also responsible for the formation of blisters at the bite site (39–41). The proteolytic action of SVMPs is also responsible for important systemic effects, such as coagulation disorders, through the cleavage of coagulation factors, which induces a procoagulant status. In addition to the protease domain and other domains such as disintegrin-like and lectin-like domains, they are also responsible for the action on platelets. The effect of the series of actions on hemostasis is characterized by the installation of consumption coagulopathy responsible for local and systemic bleeding (42, 43). Toxins are responsible for the direct stimulation of leukocytes, acting as VAMPs (venom associated molecular patterns), and acting on inflammatory components of the complement system, in addition to indirect action *via* the production of DAMPs (damage-associate molecular patterns) through proteolytic action on cellular and extracellular components (44, 45).

### Snake Venom Serine Proteases (SVSPs)

Like SVMPs, SVSPs also exert their activity as procoagulants, and target one or more coagulation factors of the blood coagulation cascade. This contributes to the digestion of prey, and affects coagulation, fibrinolysis and platelet aggregation, in addition to having an effect on the complement system and other immune system components (39, 46, 47). SVSPs have molecular masses that range between 26 and 67 kDa and present a highly conserved catalytic domain composed of the triad His57, Asp102 and Ser195 responsible for catalyzing the cleavage of peptide bonds with arginine or lysine residues at the C-terminal (47, 48). Acting as a target in the hemostatic system, SVSPs cause systemic hemodynamic disorders, specifically activating key proteins that belong to the coagulation cascade and affect systems such as the kallikrein-quinine and the complement system, and thus interfer with endothelial and platelet cells (47, 49).

### Three-Finger Toxins (3-FTxs)

The 3-FTxs compose a highly conserved family of non-enzymatic peptides, which ranges from 60 to 74 amino acid residues, and has diverse functions (24, 50). Proteomic and transcriptomic analysis of 3-FTxs has shown that these are found predominantly in Elapidae venoms, such as *Ophiophagus*, *Dendroaspis* and *Naja* venoms from Africa and Asia and *Micrurus* from the Americas, as well as in Columbridae and Hydrophiidae snake venoms (51–53). Most 3-FTx subgroups are neurotoxins, which have as their main target the cholinergic system, and present selectivity for several receptor subtypes. In the immune system, they are responsible for impairing the regulation of inflammatory processes and signaling through distinct intracellular pathways (51). Mainly, they stand out as neuromuscular blockers and are highly competitive antagonists of nicotinic acetylcholine receptors (nAChRs) in the neuromuscular junction (41). This neurotoxicity is responsible for the cardiorespiratory failure observed in snakebite patients. Bungarotoxin, isolated from *Bungarus multicinctus*, is one of the most important examples of a potent 3-FTx, and has the characteristic of high-binding to nAChRs and, as such, it is widely used as a marker in the biological studies of receptors that study the nAChRs (51). Cardiotoxins (CTXs) are considered the second largest group of 3-FTxs, and are also known as cytotoxins, due to their ability to invoke lysis in several distinct cells (51). Moreover, CTXs are acetylcholinesterase inhibitors that inhibit the enzyme at the neuromuscular junctions, thus inducing muscle spasms (51, 54). Aside from its blocking action of nAChRs, 3-FTxs are also known for several other biological responses such as inhibition of platelet aggregation, adrenoreceptors modulation, L-type calcium channel blockers and anticoagulant activity (55).

### Other Toxins

Unlike the more abundant proteins present in the venom of snakes, there are also less abundant components present in venoms with lower structural variability levels. These components add or potentiate the effects of toxins found in greater abundance, but many of them also interact with specific physiological targets (56).

In this group, there are LAAOs, HAases, nucleotidases (NUCs) PDEs. LAAOs occur in several organisms and are not exclusive to the venom of snakes in the Viperidae and Elapidae snakes (57). The concentrations of LAAO differ among snake species, thus influencing their level of toxicity. LAAOs are characterized by having a catalytic specificity for long hydrophobic and aromatic chains of amino acids, besides presenting a structural and functional variability, which causes platelet functions, disturbs plasma coagulation or leads to the death of different cell lineages (58). HAases are found in small proportions in the venom of snakes; however, they facilitate the diffusion of the venom in the tissue of the prey due to their hydrolytic characteristics of hyaluronic acid, an important component of connective tissue, thus potentiating the effects of other toxins (59, 60). NUCs have as their main function the generation of adenosine through catalytic activity, which contributes to the immobilization of prey while increasing vascular permeability, and also promotes hypotension, bradycardia and decreased locomotor activity. Among the nucleases, PDE exonucleases catalyze the hydrolysis of phosphodiester bonds, and are recognized for inducing hypotension, locomotor depression, and inhibition of platelet aggregation (61, 62). In turn, NUCs and PDEs are involved in the cleavage of nucleic acid derivatives and realigned substrates, such as ATP, ADP, and AMP. These NUCs can act independently or in synergy with other enzymes such as SVMPs, PLA_2_s, and disintegrins, which act on the inhibition of platelet aggregation and synergistically increase the anticoagulant effect of snake venoms (63–66).

## Antivenoms for Snakebite Treatment and Their Limitations

The main intervention for neutralizing toxins and reducing the effects of the envenomation is intravenous administration of snake antivenom, which is composed of polyclonal antibodies purified from the plasma of animals such as horses, goats, rabbits or sheep that have been hyperimmunized with sublethal doses of the venom (67, 68). According to the WHO (2016), by definition, antivenoms consist of a purified fraction of immunoglobulins or fragments of immunoglobulins from the plasma of animals that have been immunized against one or more snake venoms, and its administration may be limited to hospital facilities (69).

In general, antivenoms are composed of specific immunoglobulins for neutralizing snake toxins, with the IgG isotype being primarily responsible for the neutralizing activity. There are three basic formulations of antivenoms in terms of active substances. Most manufacturers produce antivenoms based on F(ab’)_2_ divalent fragments, while other antivenoms contain whole IgG molecules and a few antivenoms are based on monovalent Fab fragments (70). Depending on the type of formulation of the antivenom, there will be variations in its pharmacokinetic activity. These pharmacokinetic differences have obvious pharmacodynamic implications, for example, high distribution volumes and rapid clearance of antivenom make repeated administration necessary. One issue regarding the antivenoms therapeutic use is their limited effectiveness in reducing local damage induced by snake venoms. This has been frequently associated with the pharmacodynamic characteristics of big molecules, which results in the inability to antivenom to access affected local tissues. However, it has been shown recently that antivenom does reach the injured tissue at the site of the bite and the apparently reduced efficacy of antivenoms towards local tissue damage occurs mostly because many endogenous proinflammatory mediators are already activated by venom toxins before antivenom administration (71). In this regard, the time between the snakebite and antivenom administration is crucial for effective treatment and this is a serious problem in rural areas where access to hospitals is hampered by distance and lack of fast transport routes for the patients (72).

In the field of current therapy, the production of snake antivenoms faces some challenges, since about 70% of the antibodies produced are related to the previous antibody library of the immunized animal and do not bind directly to the venom toxins, and heterologous immunoglobulins may induce anaphylactic and pyrogenic reactions. In addition, antigenic reactivity may be reduced due to the taxonomic diversity of snakes and the different composition of venoms, according to ontogenetic or geographical variation (73). Besides these factors, it is relevant to consider that the victims with the highest risk and those most affected by envenomations are groups that live in countries and rural regions with a less favorable social and economic situation, and generally work in agriculture, fishing, hunting, forestry or are indigenous peoples. These people normally have difficulties in accessing antivenom as its distribution may be reduced in many distant regions that have a high incidence of snakebites (10, 29, 74). As a result, many patients appeal to the cultural practices or beliefs that delay the appropriate treatment and increase the risks of negative clinical outcomes. Patients make use of tourniquets, chemicals, and puncture or aspiration at the site of the bite in order to reduce the effects of the venom. These practices are common in regions where antivenoms are scarce and often impair treatment of the patient (9). Due to the difficult access to treatment, many communities have conserved their traditional methods to minimize the damage caused by snakebites (75). Medicinal plants used against snakebites are found all over the world and, as such, the use of plant extracts, leaf infusions or herbal compresses is traditionally common in many of these countries, in particular, as an effort to treat the effects of the venom such as bleeding and edema (75, 76).

Despite the various technological and scientific advances, even today, immunotherapy remains the only effective treatment against SBEs. There is no doubt that current antivenoms have been invaluable in saving lives; however, the limitations in the effectiveness of antivenom have motivated the search for alternative neutralizing agents from natural sources or synthetic compounds in order to improve or complement conventional antivenom therapy (77). Strategies have been considered that take into account the limiting aspects of antivenom and the possibility of the use of inhibitors of specific toxins, in order to aid the treatment with antivenom therapy with fast and simple interventions that could be used soon after SBEs (67, 78, 79). Therefore, the ethnobotanical knowledge of traditional communities would be a smart strategy for the discovery of molecules that are capable of neutralizing the toxins of venoms, and ancient traditional knowledge may be used for the development of alternative therapies.

The aim of the study is to identify medicinal plants and their natural products so that they can be isolated and used to inhibit the toxins from snake venom or act as an adjuvant with snake antivenoms (75, 76). Nature has been a source of essential compounds for man since time immemorial. A multitude of new molecules are constantly being discovered from diverse organisms, since it is estimated that there are about three hundred thousand species of plants distributed throughout the planet. As such, it is evident that the world’s biodiversity represents a reservoir of biological and chemical assets, which has not yet been fully exploited and, therefore, the isolation of unexplored compounds may culminate in the development of new drugs or the improvement of existing ones (80).

One of the therapeutic strategies for neutralizing the toxic components of venoms comes from the knowledge of the mechanism of action of the different types of antibodies, toxins and, above all, potent and selective enzyme inhibitors, and this strategy can aid in developing drugs with greater specificity and effectiveness (77, 81). In this context, natural products present themselves as formidable enzyme inhibitors. They are a promising source for adjuvant molecules in combination therapies and may minimize muscle damage and/or avoid tissue necrosis, prior to the patient reaching the hospital (68, 82).

Currently, about 60% of medicinal compounds are derived, or inspired by, natural products or use their pharmacophore as a model, and 75% are used in the treatment of infectious diseases (83, 84). The possibility of using natural products can lead to positive side effects in snakebites, such as a rapid administration, reduction of the diffusion and action of toxins and, therefore, a reduction in snakebite mortality and morbidity (68, 82). The continuity of studies on the mechanism of action and safety of these molecules will reveal their potential use in the development of new therapies for snakebites. This knowledge is important, and necessary, in order to improve the reality of this neglected tropical disease in many countries (84). Therefore, this review aims to provide an updated description of bioactive natural compounds isolated from plants that have been tested as potential inhibitors and help readers to understand the diversity of these compounds and their actions against snake venom, as well as presenting prospects in applications such as adjuvant inhibitors against venom toxins.

## Ethnopharmacology for Snakebite Treatment

The proposal to use natural molecules of low molecular weight is evidently not to use them as antivenom alternatives in the treatment of snakebites, but to use them as an auxiliary therapy, particularly before the administration of antivenom, in order to minimize the local effects of envenomations. A positive point about this is that some of these molecules can be used as the first therapeutic aids to be administered on the way to a health facility (79). Due to the complexity of snake venoms, it is unlikely that a single molecule will be able to neutralize all enzyme toxins, even a universal antivenom seems improbable. However, harnessing the ethnobotanical knowledge of traditional peoples so as to discover natural products, such as snake toxin inhibitors, may enable a range of new therapeutic treatments in the future.

Many studies have already been published in databases containing information on molecules that are bioactive and derived from natural products and that have evidenced antivenom activities (85). The term “natural products” can cover an extremely large and diverse variety of many chemical compounds derived and isolated from biological sources such as plants, and this interest has been widely sought for years and has always been based on the experience of randomized tests and animal test observations (68).

Plants have traditionally been used to treat snake envenomations since ancient times and are still used by many people in remote rural areas. This is due to the fact that they are easily available, are relatively inexpensive, and rarely cause complications in administration (86).

One of the ways to select bioactive species is through the traditional knowledge of the people who have been using them for generations with some degree of efficacy in various situations. For example, some plants that are being tested for their effectiveness as snake antivenom have already been used by traditional communities as treatments for snakebites, thus arousing the interest of researchers to search for their bioactive compounds (75, 76). A large part of the world’s population has already resorted to the use of popular treatments for the most diverse purposes.

Ethnobotanical and ethnopharmacological investigations have indicated hundreds of plants that are traditionally used against snakebites. Studies on about 198 species distributed in 73 botanical families have been conducted in India, which is the country with the high incidence of snakebites worldwide (87). Otero et al. (88) tested extracts of 74 plant species used by healers in the northwest region of Colombia for the treatment of snakebites and 12 of these were active against the venom of *B. atrox*. It has been shown that ethnomedicinal preparations administered orally and applied topically, such as infusions, decoctions, pulverized material and juices, can be used as antidotes in the treatment of snake envenomations (88, 89). These preparations consist mainly of leaves (48%), roots (26%) and stem barks (8%) of plants (90). Among the botanical families, Asteraceae is common in the popular use of plants against snake envenomations, and leaves of the species *Tithonia diversifolia*, *Microglossa pyrifolia* and *Conyza sumatrensis* are used in infusions (89, 91, 92).

In addition, some botanical families are highlighted due to the presence of substances with venom neutralizing properties. Fabaceae is considered the main family to have potential snake venom inhibiting substances, and is the most studied (93–103). After Fabaceae, come the families Zingiberaceae (104–108), Salicaceae (109–111) and Asteraceae (112–116).

Investigations such as these are of paramount importance for indicating the species that should be subjected to further phytochemical studies, thus enabling the discovery and development of potentially bioactive molecules. Phenolic compounds constitute the predominant group of substances that are responsible for the inhibition of snake venom. Within this group, the subclasses of flavonoids (117–120), hydroxycinnamic acids and derivatives (103, 121–125); hydroxybenzoic acids (81, 126–130), tannins (109–133), coumarins (103, 134), among others. The second majority group of substances with antivenom activity are terpenes (97, 105, 106, 112, 135–139), which are followed by alkaloids (140–146), modified glycosides (110, 111, 147), saponins (100, 101, 103, 148) and polyketides (86, 149, 150). In addition, proteins and peptides are reported as inhibitors of snake venom (61, 107, 151–153). Several substances present inhibition of the main enzyme class in abundance in most snakes, PLA_2_s, and numerous mechanisms by which these compounds can act to inhibit the venom (112, 144, 153–156).

## Plant Products as Antivenom Agents

### Alkaloids

**Figure 4:**
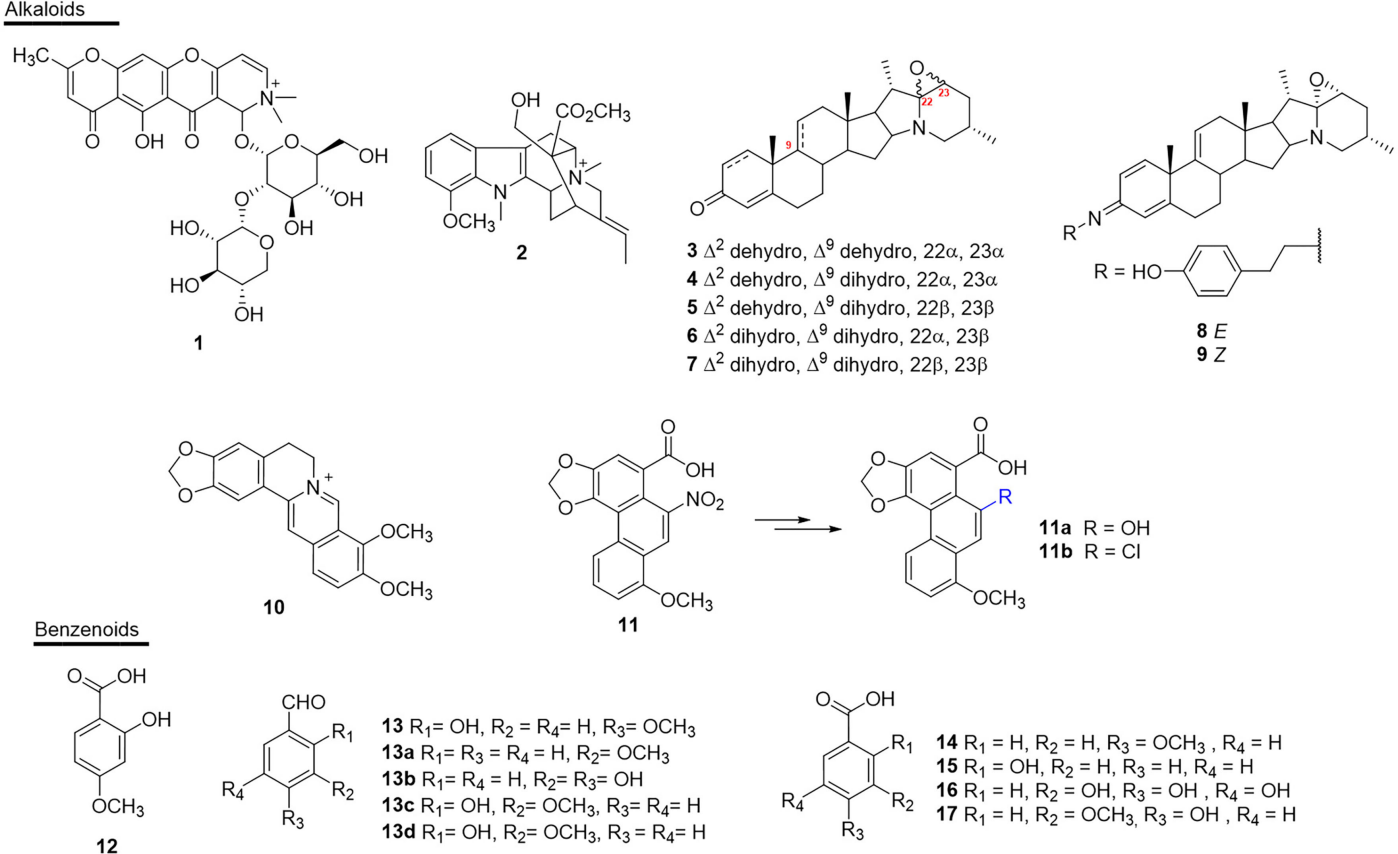
Chemical structures of snakebite treatment compounds **1-17**.

The alkaloids present numerous biological activities, but only a few reports describe their inhibitory activities against the enzymes present in snake venoms. To date only 11 active substances have been described (**Figure 4** and **Table 1**). Schumanniofoside, (**1**) a glycosilated benzopyranylpiperidinone alkaloid isolated from the stem bark of *Schumanniophyton magnificum* (Rubiaceae) in Nigeria reduced the lethal effect of *N. melanoleuca* venom *in vivo* in different concentrations (10-100 mg/kg), with the administered dose of 80 mg/kg reducing the mortality in mice by 15% (140).

**Table 1:** Alkaloids **(1–11)** and benzenoids **(12–17)** with antivenom properties.

N°	Compound	Plant	Activity inhibited	Venom or toxin	Snake	Reference
**1**	schumanniofoside	*Schumanniophyton magnificum*^TS^	lethality	Venom	*Naja melanoleuca*	(140)
**2**	12-methoxy-4-methylvoachalotine	*Tabernaemontana catharinensis*^RT^	lethality, myotoxicity	Venom	*Crotalus durissus*	(141)
**3**	22*α*,23*α*epoxy-solanida-1,4,9-trien-3-one	*Solanum campaniforme*^L^	myotoxicity, hemorrhagic and skin necrosis	Venom	*Bothrops pauloensis*	(142)
**4**	22*α*,23*α*-epoxy-solanida-1,4-dien-3-one	*S. companiforme*^L^	myotoxicity, hemorrhagic and skin necrosis	Venom	*B. pauloensis*	(142)
**5**	22*β*,23*β*-epoxy-solanida-1,4-dien-3-one	*S. companiforme*^L^	hemorrhagic, increase of creatine kinase	Venom	*B. pauloensis*	(142)
**6**	22*α*,23*α*-epoxy-solanida-4-en-3-one	*S. companiforme*^L^	hemorrhagic, necrotic, increase of creatine kinase	Venom	*B. pauloensis*	(142)
**7**	22*β*,23*β*-epoxy-solanida-4-en-3-one	*S. companiforme*^L^	necrotic, increase of creatine kinase	Venom	*B. pauloensis*	(142)
**8**	(*E*)-*N*-[8′(4-hydroxyphenyl)ethyl]-22*α*,23*α*-epoxy-solanida-1,4,9-trien-3-imine	*S. companiforme*^L^	necrotic	Venom	*B. pauloensis*	(143)
**9**	(*Z*)-*N*-[8′(4-hydroxyphenyl)ethyl]-22*α*,23*α*-epoxy-solanida-1,4-dien-3-imine	*S. companiforme*^L^	proteolytic, hemorrhagic, necrotic	Venom	*B. pauloensis*	(143)
**10**	berberine	*Cardiospermum halicacabum*^TS^	enzimatic, competitive inhibitor	PLA_2_	*Daboia russelii*	(144)
**11**	aristolochic acid	*Aristolochia indica*^R^*, A. sprucei*^S^	enzimatic, edematogenous, myotoxic, muscle damage, hemolytic	Venom, LAAO, HAase, PLA_2_, VRV-PL-VI (PLA_2_), PrTX-I (PLA_2_)	*D. russelii, D. r. pulchella, Vípera russelii, B. jararacussu, B. asper, B. pirajai, N. naja*	(145, 146, 157, 158)
**11a**	hydroxyl aristolochic acid	*A. indica*^R^	enzimatic	LAAO	*D. russelii, N. naja*	(145)
**11b**	chloride aristolochic acid	*A. indica*^R^	enzimatic	LAAO	*D. russelii, N. naja*	(145)
**12**	2-hydroxy-4-methoxy benzoic acid	*Hemidesmus indicus*^R^	hemorrhagic, edematogenous, coagulant, lethality, defibrination, inflammation	Venom	*D. russelii, V. russelii, N. kaouthia, Ophiophagus hannah, Echis carinatus*	(126, 128–130)
**13**	2-hydroxy-4-methoxy benzaldehyde	*-*	enzimatic, hemorrhagic, lethality	Venom, PLA_2_	*D. russelii, V. russelii, N. kaouthia*	(81, 127)
**13a**	3-methoxy benzaldehyde	*Janakia arayalpatra*^SS^	lethality, enzimatic	Venom, PLA_2_	*D. russelii, N. kaouthia*	(127)
**13b**	3, 4-dihydroxy benzaldehyde	*J. arayalpatra*^SS^	lethality, hemorrhagic, enzimatic	Venom, PLA_2_	*D. russelii, N. kaouthia*	(127)
**13c**	2-hydroxy-3-methoxy benzaldehyde	*J. arayalpatra*^SS^	lethality, hemorrhagic	Venom	*D. russelii, N. kaouthia*	(127)
**13d**	2-hydroxy-3-methoxybenzylalcohol	*J. arayalpatra*^SS^	enzimatic, desfibrogenation, coagulant, lethality	Venom	*D. russelii, N. kaouthia*	(127)
**14**	anisic acid	*H. indicus*^R^	lethality, defibrinogenation, hemorrhagic, edematogenous	Venom, VRV-PL-VIIIa (PLA_2_)	*V. russelii, E. carinatus, N. kaouthia, O. hannah*	(130, 160)
**15**	salicylic acid	*H. indicus*^R^	hemorrhagic	Venom	*V. russelii, E. carinatus, N. kaouthia, O. hannah*	(130)
**16**	gallic acid	*-*	proteolytic, hemorrhagic, edematogenous, dermonecrotic, myonecrotic	Venom	*D. russelii*	(161, 162)
**17**	vanillic acid	*-*	enzymatic, coagulant	5’AMP	*N. naja*	(187)

L, leaves; TS, tender shoots; R, roots; S, stem; SS, semi-synthetic; Hyaluronidase: HAase; L amino acid oxidase: LAAO; Phospholipase A_2_: PLA_2_; 5’nucleotidase: 5’AMP.

Another study reported the *in vivo* potential of 12-methoxy-4-methylvoachalotine, (**2**) an indolic alkaloid of iboga-type skeleton. This substance was isolated from the root bark of the species *Tabernaemontana catharinensis* (Apocynaceae) from Brazil (state of São Paulo) and inhibited 100% of the lethality of *C. durissus* venom when injected at the concentration of 1.7 mg/100 g, 20 seconds after injection of two mean lethal doses in mice (LD_50_) (141).

The steroidal alkaloids 22*α*,23*α*-epoxy-solanida-1,4,9-trien-3-one (**3**), 22*α*,23*α*-epoxy-solanida-1,4-dien-3-one (**4**), which were isolated from the leaves of *Solanum campaniforme* (Solanaceae) from Brazil (state of Ceará), were tested against the venom of *B. pauloensis*, and the antimyotoxic, antihemorrhagic and antinecrotizing activity was assessed after *in vitro* incubation of the venom with the extracts or isolated alkaloids (142). Through this study, it was found that the presence of alkaloids **3** and **4** resulted in a reduced necrotic area (~27.0 and 32.0-mm^2^, respectively), as well as a decreased hemorrhagic area. Subsequently, the same research group studied the other constituents of the same plant and found four new steroidal alkaloids with biological activity against the venom of *B. pauloensis*: 22*β*,23*β*-epoxy-solanida-1,4-dien-3-one (**5**), 22*α*,23*α*-epoxy-solanida-4-en-3-one (**6**), 22*β*,23*β*-epoxy-solanida-4-en-3-one (**7**), (*E*)-*N*-[8’(4-hydroxyphenyl)ethyl]-22*α*,23-*α*-epoxy-solanida-1,4,9-trien-3-imine (**8**) and (*Z*)-*N*-[8′(4-hydroxyphenyl)ethyl]-22*α*,23*α*-epoxy-solanida-1,4-dien-3-imine (**9**). In the assays, compounds **3, 5, 6** and **9** showed antihemorrhagic activity, while compounds **6, 7, 8** and **9** showed antinecrotic activity (143).

The isoquinoline alkaloid berberine (**10**), obtained from the species *Cardiospermum halicacabum* (Sapindaceae) from India, was discovered to be a PLA_2_s inhibitor of the venom of *D. russelii*. The activity was characterized through the incubation of the plant extract in the process of crystallization of the toxin, which allowed the co-crystallization of PLA_2_s with berberine and, consequently, the discovery of this natural product as an anti-inflammatory substance of interest for treating snakebites (144).

The nitrophenanthrene carboxylic acid alkaloid aristolochic acid (**11**), found in species of *Aristolochia* (Aristolochiaceae) from India, has been reported as a promising antivenom agent. Previous studies report *in vitro* inhibitory activity against LAAO of *D. russelii* (19% inhibition- IC_50_ = 33.6 µM) (145). It has also been shown that, in the venom of *D. russelii*, **11** inhibited (0.16 µM) the edema-inducing activity of the enzyme by 50% (146). Another study showed that the pre-incubation of the complex obtained from this compound (13.7 µg/mL) reduced the myotoxic effects of piratoxin-I (PrTX-I) in the venom of *B. pirajai* (157).

However, the ability of **11** to bind to DNA is related to its carcinogenesis, which makes it impossible to use this compound (145). On the other hand, semisynthetic derivatives obtained from **11** by replacing the nitro group with a chlorine atom (**11a**) or a hydroxyl group (**11b**) eliminated the interaction with DNA, while achieving inhibition of the LAAO enzyme from the venom of *D. russeli*. In addition, substance **11a** presented greater capacity of reduced LAAO-induced reactive oxygen species (ROS) generation in two cells: human embryonic kidney cells (HEK293) (76%) and human hepatocellular carcinoma (HepG2) (68%). Substance **11b** also showed significant inhibition of ROS generation, which was induced by LAAO. Additionally, cellular viability determined through the redox potential for **11a** and **11b** presented ~86 and ~67%, respectively, for HEK293, and ~74 and ~70%, respectively, for HepG2 (145).

Studies with structural adaptations of natural inhibitors, such as molecular docking, have been conducted to understand the interaction profile of natural inhibitors by binding to active sites of the toxin and have helped to elucidate the inhibition abilities and aided in the design of molecular modifications and adaptations in conformations to improve their inhibitory effect (158, 159).

### Benzenoids

A number of simple substances with substituted benzene nuclei have been reported in some plants and may be potential snake venom inhibitory molecules (**Figure 4** and **Table 1**). Among these plants, *H. indicus* (Apocynaceae), which is endemic to India, was the target of studies that showed the potential of 2-hydroxy-4-methoxy benzoic acid (**12**) against the venom of *D. ruselii*, *N. kaouthia*, *O. hannah* and *E. carinatus* (126, 128–130). This substance effectively inhibited the activity of the venom, reducing its lethal, hemorrhagic, and coagulant effects, as well as effectively neutralizing the inflammation induced by *D. russelii* venom in rodents. Another benzenoid isolated from this plant species, 2-hydroxy-4-methoxy benzaldehyde (**13**), showed neutralizing activity of PLA_2_s (*in vitro*) and reduced lethality and hemorrhagic activity induced by the venom of *D. russelii* (81).

Compound **13** was also obtained from the species *Janakia arayalpatra* (Periplocaceae) from India (Jammu) in considerable quantities, which led the authors to prepare semi-synthetic derivatives (**13a**-**13d**). These compounds were active against the venom of *D. russelii* and in *in vivo* tests neutralized the hemorrhagic effect, lethality and PLA_2_s activity induced by the venom. Additionally, in the same study, all compounds showed neutralization of lethality and hemorrhagic activity of *N. kaouthia* venom (127).

Another study showed the inhibitory effect of the compounds anisic acid (**14**) and salicylic acid (**15**), also from *H. indicus*, against the venoms of *D. russelii, E. carinatus*, *N. kaouthia* and *O. hannah*. The lethal effect of the venom and defibrinogenation were 100% neutralized by 14, both in *in vitro* and *in vivo* studies. Hemorrhagic activity was 100% neutralized by **15** (130). However, the exact mechanisms of the neutralization of the venom by these chemical compounds have not yet been established (130, 160).

Gallic acid (**16**) inhibited the *in vitro* proteolytic activity (IC_50_ 0.58 µM) of the venom of *D. russelii*, but did not inhibit the PLA_2_s activity of the same venom (161). However, the enzymatic inhibitory activity of PLA_2_s (63%) and inhibition of cytotoxicity induced by PLA_2_s (~78%) in *C. durissus* venom was demonstrated in the study by Pereañez et al. (162). Compound **16** was also isolated from *Anacardium humile* (Anacardiaceae) from Brazil (Minas Gerais) and was able to inhibit the myotoxic activity induced by the raw venom of *B. jararacussu* and its two main myotoxins, bothropstoxin I and II (BthTX-I and BthTX-II). In addition, compound 16 also inhibited the hemorrhagic effect (IC_50_ 0.2 µM) and edema formation (IC_50_ 2 µM) in *in vivo* experiments (161).

In addition, vanillic acid (**17**) selectively and specifically inhibited the enzymatic activity of 5’nucleotidase (5’AMP), which is known to affect hemostasis by inhibiting platelet aggregation among other enzymes present in the venom of *N. naja*. In a dose-dependent manner, compound **17** inhibited the anticoagulant effect of *N. naja* venom by up to 40%. Inhibition studies with **17** suggest that 5’AMP probably interacts with one or more factors of the intrinsic blood clotting pathway to cause the anticoagulant effect (154).

### Hydroxycinnamic Acids and Derivatives

**Figure 5:**
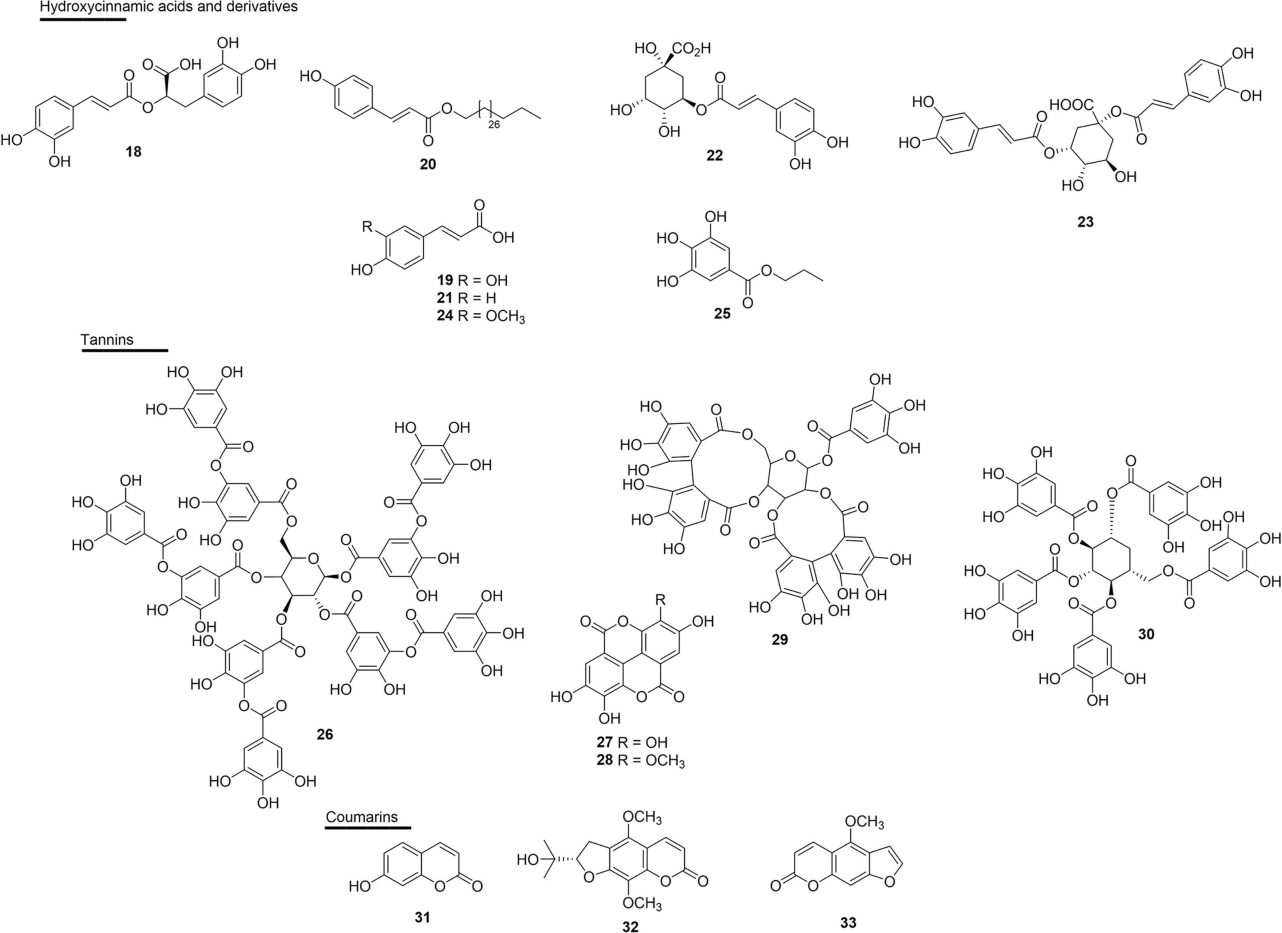
Chemical structures of snakebite treatment compounds **18-33**.

Rosmarinic acid (**18**), a esterefied hydroxycinnamic acid isolated from several plants, has well-established antivenom properties. Compound **18** isolated from *Cordia verbenacea* (Boraginaceae) inhibited edema and myotoxic activity induced by basic PLA_2_s: BthTX-I and BthTX-II. This molecule also almost completely inhibited the myotoxic effects and partially inhibited edema induced by both basic PLA_2_s, thus giving the idea of dissociation between the catalytic and pharmacological domains (125). The effect of **18** has also been shown against the venoms of *Trimeresurus flavoviridis, Gloydius blomhoffii*, *Bitis arietans, C. atrox, Agkistrodon bilineatus* and *Deinagkistrodon acutus* (122–124) (**Figure 5** and **Table 2**).

**Table 2:** Hydroxycinnamic acids **(18–25)** and tannins **(26–33)** with antivenom properties.

N°	Compound	Plant	Activity inhibited	Venom or toxin	Snake	Reference
**18**	rosmarinic acid	*Cordia verbenacea*^L^*, Argusia argentea*^L^	enzimatic, edematogenous, myotoxicity, hemorrhagic, hidrolytic fibrogenolysis	Venom, BthTX-I, BthTX-II (PLA_2_), SVMP	*B. jararacussu, Trimeresurus flavoviridis, Gloydius blomhoffii, Bitis arietans, C. atrox, Agkistrodon bilineatus, Deinagkistrodon acutus, Protobothrops flavoviridis*	(122–125)
**19**	caffeic acid	*-*	reduction in plasma fibrogen, myotoxic, muscle damage, cytotoxicity	Venom, SVMP, PrTX-I (PLA_2_)	*B. pirajai, C. d. cumanensis*	(157, 162)
**20**	triacontyl *p*-coumarate (PCT)	*Bombacopsis glabra*^RB^	reduction in plasma fibrogen, coagulant, myotoxicity	Venom, SVMP	*Bothropoides pauloensis*	(121)
**21**	*p*-coumaric acid	*-*	enzimatic	PLA_2_	*D. r. pulchella*	(156)
**22**	chlorogenic acid	*Vernonia condensata*	lethality, enzimatic	Venom, PLA_2_	*B. jararaca, D. russelii*	(103)
**23**	cynarin	*Cynara scolymus*	lethality	Venom	*B. jararaca*	(103)
**24**	ferulic acid	*Baccharis uncinella*^A^	enzimatic, edematogenous, cytotoxicity	PLA_2_	*C. d. terrificus, C. d. cumanensis*	(112, 162)
**25**	propylgallate	*-*	enzimatic, cytotoxicity, myotoxicity	Venom	*C. d. cumanensis*	(162)
**26**	tannic acid	*-*	enzimatic, hemorrhagic, lethality, creatine kinase reduction	Venom, HAase	*C. adamenteus*	(120)
**27**	ellagic acid	*Casearia sylvestris*^L^	enzimatic, edematogenous, myotoxicity	Venom, PLA_2_	*B. jararacussu*	(109)
**28**	3`-*O*-methyl ellagic acid	*C. sylvestris*^L^	edematogenous, myotoxicity	Venom, PLA_2_	*B. jararacussu*	(109)
**29**	casuarictin	*Laguncularia racemosa*^L^	edematogenous, myonecrosis	PLA_2_	*C. d. terrificus*	(131)
**30**	pentagalloylglucopyranose	*Mangifera indica*^SK^	enzimatic	PLA_2_, HAase, LAAO	*Calloselasma rhodostoma, N. n. kaouthia*	(133)
**31**	umbelliferone	*-*	edematogenous, inflammatory, platelet aggregation	Venom, PLA_2_	*B. neuwiedi*	(163)
**32**	(+)-alternamin	*Murraya alternans*^A^	hemorrhagic	Venom	*T. flavoviridis*	(134)
**33**	bergapten	*Dorstenia brasiliensis*	lethality	Venom	*B. jararaca*	(103)

L, leaves; RB, root bark; A, aerial; SK, seed kernels. Hyaluronidase: HAase; L amino acid oxidase: LAAO; Phospholipase A_2_: PLA_2_; Metalloproteinase: SVMP.

Evidence of the potential of caffeic acid (**19**) was obtained by complexation with piratoxin-I, a PLA_2_ containing lysine as a residue at position 49 (PLA_2_s-Lys49) of the venom of *B. pirajai*, and resulted in the partial neutralization of the myotoxic activity of PrTX-I (157). This substance also inhibited 41% the cytotoxic activity induced by *C. durissus* PLA_2_s (162).

The modified derivative triacontyl *p*-coumarate (**20**), which was isolated from *Bombacopsis glabra* (Bombacaceae) from Brazil (state of Bahia), was promising against the harmful effects of *B. pauloensis* venom and also against isolated SVMPs (jararhagin) or PLA_2_s (BnSp-6) (121). Compound **20** neutralized fibrinogenolytic activity and plasma fibrinogen depletion (53%) when induced by venom or isolated toxins. This molecule also efficiently inhibited hemorrhagic activity (3 MDH) and jararhagin-induced hemorrhagic activity (121) (**Figure 5** and **Table 2**).

The substance *p*-coumaric acid (**21**) complexed with PLA_2_s from *D. russelii* showed effective catalytic inhibitory activity with an IC_50_ of 38.0 µM (156). Chlorogenic acid (**22**) and cynarin (**23**) showed percentages of inhibition of mortality against *B. jararaca* venom in mice with high (90%, **22**) and weak (30%, **23**) activities, respectively (103).

Ferulic acid (**24**) showed the potential to inhibit PLA_2_s, as well as a strong inhibitory activity against the induction of edema by the PLA_2_s enzyme of *C. durissus* (112). Another study reported the enzymatic inhibitory activity of PLA_2_s (17%) in this substance, as well as the inhibition of cytotoxicity induced by PLA_2_s (37%) in the venom of *C. durissus* (162). In this same study, the inhibitory capacity of propylgallate (**25**) in the enzymatic (51%) and cytotoxic (94%) activity of PLA_2_s of the venom of this same species were also reported (**Figure 5** and **Table 2**).

### Tannins

Some studies of tannins as active molecules against snake venoms have been reported. Gallotannin tannic acid (**26**), found in several plants, but obtained commercially, efficiently inhibited the HAases and the hemorrhagic effect, and reduced the *in vivo* lethal effect of *C. adamanteus* venom, causing an increase in the survival time of mice (120). Ellagitannins were isolated from *Casearia sylvestris* (Salicaceae) leaves from Brazil (state of São Paulo), and ellagic acid (**27**) and 3’-*O*-methyl ellagic acid (**28**) were tested against the effects of venom and PLA_2_s (Asp 49 BthTX-II) from the venom of *B. jararacussu*. The inhibition constant (Ki) values for enzymatic activity were approximately **3** and **7** nM, for **27** and **28**, respectively; moreover, the IC_50_ values found in the edematogenic and myotoxic activity were 23.8 µM for **27** and **34** µM for **28** (109) (**Figure 5** and **Table 2**).

Casuarictin (**29**), an ellagitannin isolated from the leaves of *Laguncularia racemosa* (Combretaceae) from Brazil (state of São Paulo), was evaluated in PLA_2_s isolated from the rattlesnake *C. durissus*. The compound was able to form a protein complex consisting of a stable complex of PLA_2_s and casuarictin (casu). In addition, molecular interactions of casu with PLA_2_s were able to virtually eliminate the native edematogenic effect, as well as protein-induced myonecrosis when injected 10 min after PLA_2_s. Therefore, casu can be considered to be a potential anti-inflammatory substance that can be used to treat PLA_2_s-induced edema and myonecrosis (131). Another study performed with the ethanolic extract of the seed grains of Thai mango (*Mangifera indica* - Anacardiaceae) and its main active ingredient, and 1,2,3,4,6-pentagalloyl glucopyranose (**30**) exhibited inhibitory effects on the enzymatic activities of PLA_2_s, HAases and LAAO of the venoms from *Calloselasma rhodostoma* and *N. naja* in *in vitro* tests (133) (**Figure 5** and **Table 2**).

### Coumarins

The incubation of Bn IV, a Lys49 PLA_2_s from *B. neuwiedi* venom together with coumarin umbelliferone (**31**), a substance abundant in *Citrus* spp., virtually eliminated platelet aggregation, edema (IC_50_ 0.2 µM) and myotoxicity induced by Bn IV, and also decreased its inflammatory effects. Compound **31** showed interaction with Asp and Lys residues from the PLA_2_s catalytic site, which are interactions that are important for the activity of the toxin (163). In the class of coumarins, dihydrofuranocoumarin (+)-alternamin (**32**), a new substance extracted from the aerial parts of *Murraya alternans* (Rutaceae) from Myanmar, was able to inhibit bleeding induced by the venom of *T. flavoviridis* by 24% at the concentration of 250 µg/mL when compared to the control (134). Also joining this list, bergapten (**33**), isolated from *Dorstenia brasiliensis* (Moraceae) from Brazil (state of Rio de Janeiro), was modest in its inhibition of the lethality of *B. jararaca* venom in mice (20% reduction in lethality) (103) (**Figure 5** and **Table 2**).

### Flavonoids

**Figure 6:**
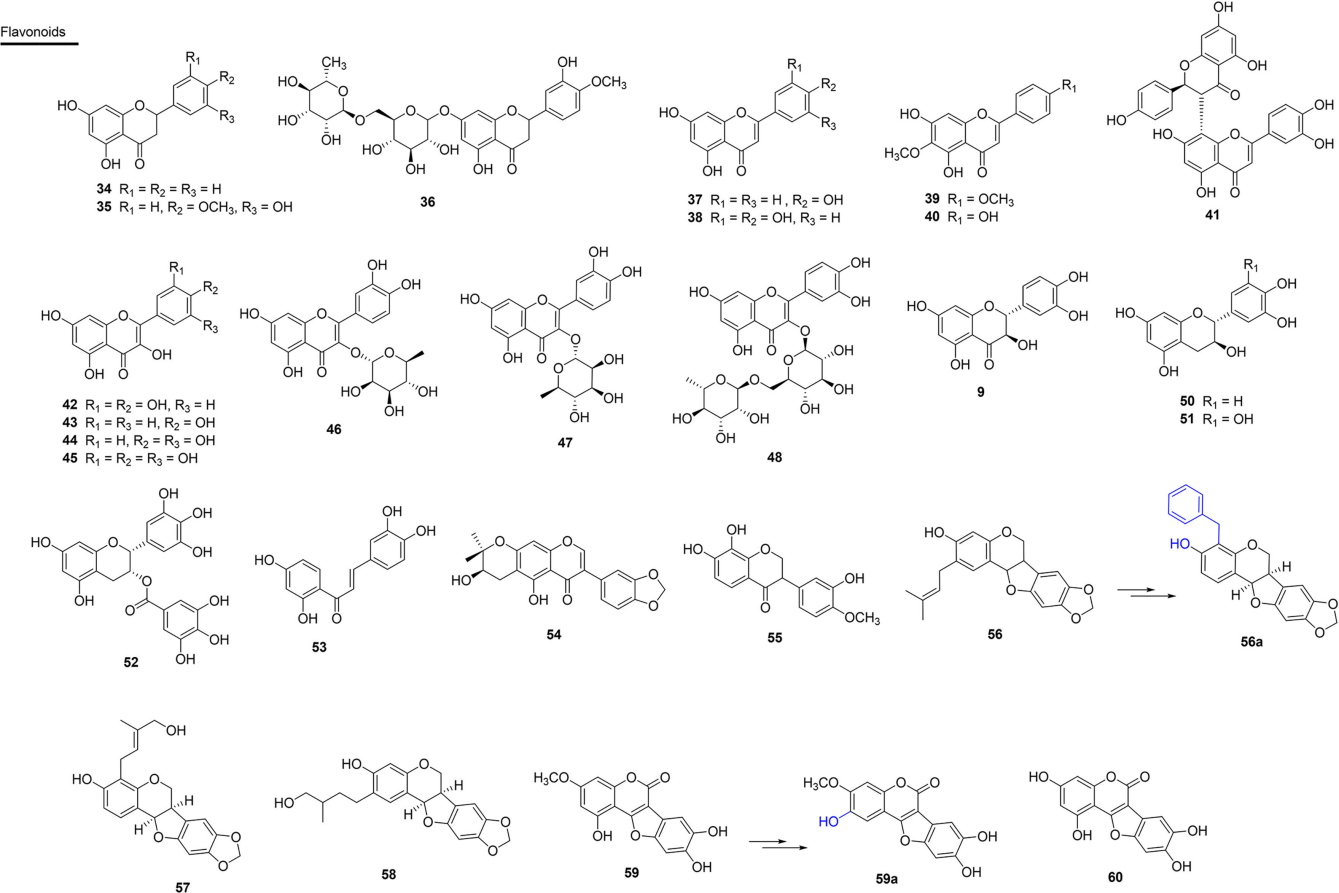
Chemical structures of snakebite treatment compounds **34-60**.

Flavonoids have numerous biological activities, among them, anti-inflammatory and antioxidant activities stand out, and this makes several representatives of this class potential antivenom agents. In this context, two SVSPs, thrombin-like SVSPs (SP1 and SP2), of *C. simus* venom were isolated and incubated *in vitro* with the flavanone aglycon hesperetin (**34**), which is commonly isolated in Rutaceae. The results indicated that **34** acts as a potent non-competitive inhibitor (against SP1) or mixed inhibitor (against SP2). Thus, a naturally occurring flavone that can be easily extracted from oranges can serve as a low-cost inhibitor of the investigated snake venom proteases (118). Substance **34** also inhibits PLA_2_s activity of *C. atrox* venom (164). Additionally, substance **34**, which was obtained from orange peels, acted as a reversible inhibitor of SVSP isolated from the venom of *B. jararaca* (165) (**Figure 6** and **Table 3**).

**Table 3:** Flavonoids **(34-53)** and isoflavonoids and derivates **(54-60)** with antivenom properties.

N°	Compound	Plant	Activity inhibited	Venom or toxin	Snake	Reference
**34**	hesperetin	*Citrus sinensis*^P^	enzimatic	SVSP	*C. simus*	(118, 165)
**35**	pinostrobin	*Renealmia alpinia*^L^	enzimatic, myotoxicity, proteolytic, hemolytic, coagulant	Venom, PLA_2_	*C. d. cumanensis, B. asper*	(104)
**36**	hesperidin	*C. sinensis*	enzimatic, hemorrhagic, lethality	Venom, HAase	*C. adamenteus*	(103)
**37**	apigenin	*-*	enzimatic, hemorrhagic, lethality	Venom, HAase	*C. adamenteus, C. atrox, N. n. sputatrix*	(119, 120, 164)
**38**	luteolin	*-*	enzimatic, hemorrhagic, lethality	Venom, HAase	*C. adamenteus, C. atrox, N. n. sputatrix*	(119)
**39**	pectolinarigenin	*A. integrifolia*^L^	enzimatic	PLA_2_, HAase	*B. atrox*	(166)
**40**	hispidulin	*A. integrifolia*^L^	enzimatic	PLA_2_, HAase	*B. atrox*	(166)
**41**	morelloflavone	*Garcinia madruno*^A^	enzimatic, coagulant, myotoxicity, edematogenous	PLA_2_	*C. d. cumanensis*	(167)
**42**	quercetin	*Phyllanthus klotzschianus*^A^*, Morus nigra*^L^*, Erythroxylum ovalifolium*^L^*, E. subsessile*^S^	enzimatic, edematogenous, proteolytic, lethality	Venom, HAase	*B. jararacussu, Lachesis muta, B. jararaca, N. naja*	(104, 117, 168, 171)
**43**	kaempferol	*-*	enzimatic, hemorrhagic, lethality	Venom, HAase	*C. adamanteus, C. atrox, N. n. sputatrix*	(119, 120)
**44**	fisetin	-	enzimatic	PLA_2_	*C. atrox*	(164)
**45**	myricetin	-	enzimatic, proteolytic, hemorrhagic	Venom, PLA_2_	*C. atrox, B. atrox*	(164, 172)
**46**	quercitrin	-	enzimatic	PLA_2_	*C. atrox*	(164)
**47**	quercetin-3-*O*-rhamnoside	*Euphorbia hirta*^WP^	enzimatic, hemolytic, lethality, edematogenous	Venom, PLA_2_, HAase	*N. naja*	(173)
**48**	rutin	*E. ovalifolium*^S^*, E. subsessile*^S^	hemorrhagic	Venom	*L. muta*	(164, 168)
**49**	taxifolin	-	enzimatic	PLA_2_	*C. atrox*	(164)
**50**	catechin	*Scolopia chinensis*^S^	enzimatic	PDE-I	*-*	(110)
**51**	gallocatechin	*Schizolobium parahyba*^L^	hemorrhagic, fibrogenolytic, myotoxicity	Venom, SVMP, PLA_2_	*B. jararacussu, B. neuwiedi, B. alternatus*	(93)
**52**	epigallotechin gallate	*-*	enzimatic, cytotoxicity	PLA_2_	*C. d. cumanensis*	(162)
**53**	butein	*Butea monosperma*	enzimatic	Daboxina (PLA_2_)	*D. russelii*	(174)
**54**	harpalycin 2	*Harpalyce brasiliana*^L^	enzimatic, edematogenous, myotoxicity	Venom, PLA_2_, PrTX-III	*B. pirajai*	(95, 96)
**55**	7,8,3’-trihydroxy-4’-methoxyisoflavone	*Dipteryx alata*^S^	myotoxicity, neuromuscular	Venom, BthTX-I	*B. jararacussu*	(98)
**56**	edunol	*Brongniartia podalyrioides*^R^*, H. brasiliana*^R^	morthality, myotoxicity, proteolytic, enzimatic	Venom, PLA_2_	*B. atrox, B. jararacussu*	(99, 102)
**56a**	bioisostere	*H. brasiliana*^SS^	myotoxicity	Venom	*B. jararacussu*	(99)
**57**	cabenegrins A-I	*Annona crassiflora*^R^	lethality	Venom	*B. atrox*	(176)
**58**	cabenegrins A-II	*A. crassiflora*^R^	lethality	Venom	*B. atrox*	(176)
**59**	wedelolactone	*Eclipta prostrate*^SY^	proteolytic, myotoxicity	Venom, PLA_2_	*B. jararacussu*	(66)
**59a**	analogue of wedelolactone	*E. prostrate*^SY^	myotoxicity	Venom	*B. jararacussu*	(66)
**60**	demethylwedelolactone	*E. alba*^R^	myotoxicity	PLA_2_	*C. d. terrificus, B. jararacussu*	(177)

L, leaves; R, roots; S, stem; SS, semi-synthetic; A, aerial; P, peels; SY, synthesis. Hyaluronidase, HAase; Phosphodiesterase I, PDE-I; Phospholipase A_2_, PLA_2_; Metalloproteinase, SVMP; Serine protease, SVSP.

The flavanone pinostrobin (**35**) was isolated from the leaves of *Renealmia alpinia* (Zingiberaceae) from Colombia. It is a plant used in folk medicine to treat snake bites and was evaluated as to its ability against the venom of *C. durissus* and *B. asper*. Compound **35** presented an IC_50_ of 1.76 µM against PLA_2_s activity in *C. durissus* venom. When mice were injected with PLA_2_s and treatments of 0.4, 2.0 and 4.0 µM of pinostrobin were applied, PLA_2_s-induced myotoxic activity was inhibited by up to 87% (104). Compound **35** was effective in inhibiting proteolytic effects (22%) induced by *B. asper* venom, and presented indirect percentage inhibition of hemolytic activity of 21%. In this same study, *R. alpinia* extract inhibited indirect hemolytic, coagulant and proteolytic activities of *B. asper* venom after pre-incubation *in vitro* (**Figure 6** and **Table 3**).

The glycosilated flavanone, hesperidin (**36**), which was isolated from *Citrus sinensis* (Rutaceae), showed moderate inhibitory action of lethality caused by *B. jararaca* venom (103). Flavones, such as the aglycones apigenin (**37**) and luteolin (**38**) of synthetic origin, are inhibitors of the hyaluronidase and hemorrhagic action and reduces of the lethality of the venom of *C. adamenteus* (119, 120). Additionally, the synthetic compound **37** showed PLA_2_s inhibitory activity of *C. atrox* venom (164). The flavones pectolinarigenine (**39**) and hispiduline (**40**), isolated from *A. integrifolia* (Verbenaceae) from Brazil (state of Roraima), partially inhibited the PLA_2_s activities (20 and 15% respectively) and HAases in *B. atrox* venom with 60 and 40% inhibition, respectively (166) (**Figure 6** and **Table 3**).

Another prominent representative is morelloflavone (**41**), which is a dimeric flavone isolated from *Garcinia madruno* (Clusiaceae) from Colombia. This compound exhibited IC_50_ values of 0.48 µM and 0.38 µM in PLA_2_s enzymatic activity for aggregate and monodisperse substrates, respectively. Results of molecular docking with **41** suggest the formation of hydrogen bonds with the residues Gly33, Asp49, Gly53 and Thr68 of the toxin, which are fundamental for inhibition (167) (**Figure 6** and **Table 3**).

Flavonols are among the most studied analogues, especially because they are more recurrent and often isolated in phytochemical approaches. Aglycone quercetin (**42**), isolated from *Morus nigra* (Moraceae) (117), *Phyllanthus klotzschianus* (Phyllanthaceae) (103) and *Erythroxylum ovalifolium* (Erythroxylaceae) (168), have been shown to be potent inhibitors of edema, proteolytic activity and lethality induced by the venom of the snakes *B. jararacussu*, *L. muta* and *B. jararaca*, respectively. Compound **42**, of synthetic origin, was also able to inhibit the PLA_2_s activity of *D. russelli* (IC_50_ 2 µM) and *N. naja* venoms (maximum inhibition of 40%) (169). The results obtained in the study of Cotrim et al. (2011) showed the potential of **42** to inhibit the PLA_2_s activity of *D. russelii* venom, as well as inhibiting *C. durissus* venom-induced platelet-aggregation and myotoxicity by approximately 40%. This compound completely inhibited the activity of purified HAases from the venom of *N. naja* (170, 171) (**Figure 6** and **Table 3**).

In addition to this, kaempferol (**43**) showed antivenom potential (120), as well as the flavonoids fisetin (**44**) and myricetin (**45**) of synthetic origin, which presented inhibitory potential against the PLA_2_s of *C. atrox* venom. Compound **4**5 was also active, exhibiting an IC_50_ value of 150 µM and 1 µM for inhibition of *B. atrox* venom proteolytic and hemorrhagic activities, respectively (172). In the same study, the glycosilated flavonol quercitrin (**46**), which is of synthetic origin, also showed inhibitory potential against PLA_2_s of *C. atrox* venom. In addition, compound **46**, isolated from the leaves of *Brownea rosa-de-monte* (Fabaceae) from Panama, showed high inhibition of the coagulant and hemorrhagic effects of the venom of *B. asper*. Furthermore, a 0.1 µM concentration of **43** extended the plasma coagulation time by two to six times (94).

Another glycosilated flavonol, quercetin-3-*O*-rhamnoside (**47**), isolated from the species *Euphorbia hirta* (Euphorbiaceae) from India, significantly inhibited (93%) *N. naja* PLA_2_s evaluated *in vitro* using egg yolk as a substrate, and also inhibited HAases and hemolytic activity (173). In addition, edema and lethality were reduced, prolonging the lifespan of the mice (173). Rutin (**48**) inhibited the hemorrhagic activity of *L. muta* venom *in vivo* by 28% (168) and also showed inhibitory activity (40%) of PLA_2_s of *C. atrox* venom (164) (**Figure 6** and **Table 3**).

Taxifolin (**49**), a flavanonol of synthetic origin, exhibited potential for inhibition of the PLA_2_s enzyme of *C. atrox* venom (164). Some papers have already reported flavan-3-ols as potential antivenom substances. Flavan-3-ol catechin (**50**), isolated from the stem of *Scolopia chinensis* (Salicaceae) from China, also showed inhibitory activity (16%) against snake venom phosphodiesterase I (PDE I) (110). This substance also showed PLA_2_s inhibitory activity of *C. atrox* venom, and is of synthetic origin (164). Flavan-3-ol esterified with gallic acid, gallocatechin (**51**), isolated from the leaf extract of *Schizolobium parahyba* (Fabaceae) from Brazil (state of Minas Gerais), neutralized the biological and enzymatic activities of venoms and toxins isolated from *B. jararacussu* and *B. neuwiedi* (93). Compound **51** exhibits efficient inhibition of hemorrhagic and fibrinogenolytic activity of isolated SVMPs (Bjussu-MP-I, Bjussu-MP-II). Gallocatechin also inhibited the myotoxic activity of *B. alternatus* venom and BnSP-6 (Lys49 PLA_2_s of *B. neuwiedi*) (93). Epigallotechin gallate (**52**), abundant in *Ilex paraguariensis* (yerba mate), was commercially acquired and evaluated for its PLA_2_s enzymatic inhibition ability *in vitro* using egg yolk as a substrate. In addition, the compound decreased the cytotoxic effect induced by a myotoxic PLA_2_s *in vitro* of the venom of *C. durissus*, with an inhibitory activity (IC_50_ = 0.38 µM). Results show that **52** is considered a potential antmyotoxic agent (162).

Chalcone butein (**53**), isolated from *Butea monosperma* (Fabaceae), inhibited the activity of daboxin P, a PLA_2_s, with an IC_50_ value of 541 µM. In addition, this substance inhibited the PLA_2_s activity of the raw venom (5 µg/ml) of *N. naja* (100%), *B. caeruleus* (49%), *D. russelii* (72%) and *E. carinatus* (47%) at a concentration of 1,200 µM (174) (**Figure 6** and **Table 3**).

### Isoflavonoids and Derivatives

When analyzing the effect of the isoflavone harpalycin 2 (**54**), isolated from the leaves of *Harpalyce brasiliana* (Fabaceae) from Brazil (state of Ceará) and used in folk medicine as an anti-inflammatory for the treatment of snakebites, promising activities were found. Compound **54** inhibited the enzymatic activity and edematogenic and myotoxic effects of PLA_2_s from *B. pirajai*, *C. durissus* and *N. naja* venoms. Piratoxin 3 (PrTX-III) (*B. pirajai* venom) was inhibited by 59%, *C. durissus* venom by 79% and *N. naja* venom by 88%. Edema in mouse paws induced by exogenous administration of PLA_2_s showed significant inhibition by harpalycin 2 (Har2) in the initial stage. In addition, Har2 also inhibited the myotoxic activity of these PLA_2_s (95, 96).

The compound 7,8,3’-trihydroxy-4’-methoxyisoflavone (**55**), isolated from *Dipteryx alata* (Fabaceae) from Brazil (Tocantins), was able to neutralize neurotoxicity (in phrenic nerve-diaphragm experiments in mice) and myotoxicity against the venom of *B. jararacussu* (98). Pre-incubation of **55** (200 µg/mL) with the venom attenuated the induced neuromuscular blockade by 84%. The neuromuscular blockade caused by BthTX-I, the main myotoxic PLA_2_s of this venom, was also attenuated by **55**. Histological analysis of the diaphragm muscle incubated with **55** showed that most of the fibers were preserved (only 9% were damaged) when compared to the venom on its own (50%) (98).

The derivatives of isoflavonoids, pterocarpans and coumestans, have also been identified as antivenom agents. Pterocarpan edunol (**56**), isolated from the root of *Brongniartia podalyrioides* (Fabaceae) from Mexico, reduced the lethality of *B. atrox* venom by 30% after administration of 3.1 mg/kg in mice that were previously treated by the same route with an LD_50_ of the venom (102). Substance **56** was also isolated from the root of *Harpalyce brasiliana* (Fabaceae) from Brazil. It was obtained by synthesis in order to obtain larger amounts of material for the tests and also showed antimyotoxic, antiproteolytic and anti-PLA_2_s properties (175). These properties could be enhanced by the synthesis of a derivative of **56**, the bioisostere (**56a**), in which the prenyl group was replaced by the benzyl group. Compound **56a** was able to fully inhibit the myotoxic activity of the venom by pre-incubation *in vitro* with an IC_50_ of 9.97 µM. Interestingly, at 100 mM, this pterocarpan also inhibited 65% of the phospholipase activity of the venom from *B. jararacussu*, as well as more than 80% of its proteolytic activity (175).

Other prenylated pterocarps in ring A are described as very active compounds against *B. atrox* venom, with cabenegrins A-I (**57**) and A-II (**58**) being potential lead compounds (176). These substances were isolated from the plant *Annona crassiflora* (Annonaceae), which is a popular medicinal plant from northeastern Brazil that is used for treating snakebite (176) (**Figure 6** and **Table 3**).

The coumestan, wedelolactone (**59**) has been isolated from species such as *Eclipta alba* and *E. prostrate* (Asteraceae) in Brazil (state of São Paulo) (115, 116, 177). In the study by Diogo et al. (2009), this compound inhibited basic PLA_2_s-induced myotoxic activity from the venoms of *C. durissus* and *B. jararacussu* (BthTX-I and II) (177). Compound **59** also inhibited the proteolytic, PLA_2_s and myotoxic activity (IC_50_ = 1 µM) of the venom of *B. jararacussu* (178).

Active at 30 µM, the analogue of wedelolactone (**59a**), which was synthesized with different patterns of oxygenation in the A and D rings, antagonized the release of creatine kinase (CK) induced by the venom of *B. jararacussu* in skeletal muscle. Compound **59a** also inhibited the myotoxic activity of the venom with an IC_50_ of 1 µM, which is similar to that of the wedelolactone compound (178). In addition, compound **59a** was shown to be less potent for binding to benzodiazepine receptors, indicating that **59a** is less susceptible to producing adverse effects in the central nervous system (178). Given the results, it is possible that these products may be useful in the therapy of snakebite and other coagulation disorders (178). Another study proved the inhibitory effect of the compound demethylwedelolactone (**60**) from *E. alba*, in which it neutralized the myotoxic activity induced by isolated PLA_2_s (BthTX-I and II) from *C. durissus* and *B. jararacussu* venom (177) (**Figure 6** and **Table 3**).

### Modified Glycosides

**Figure 7:**
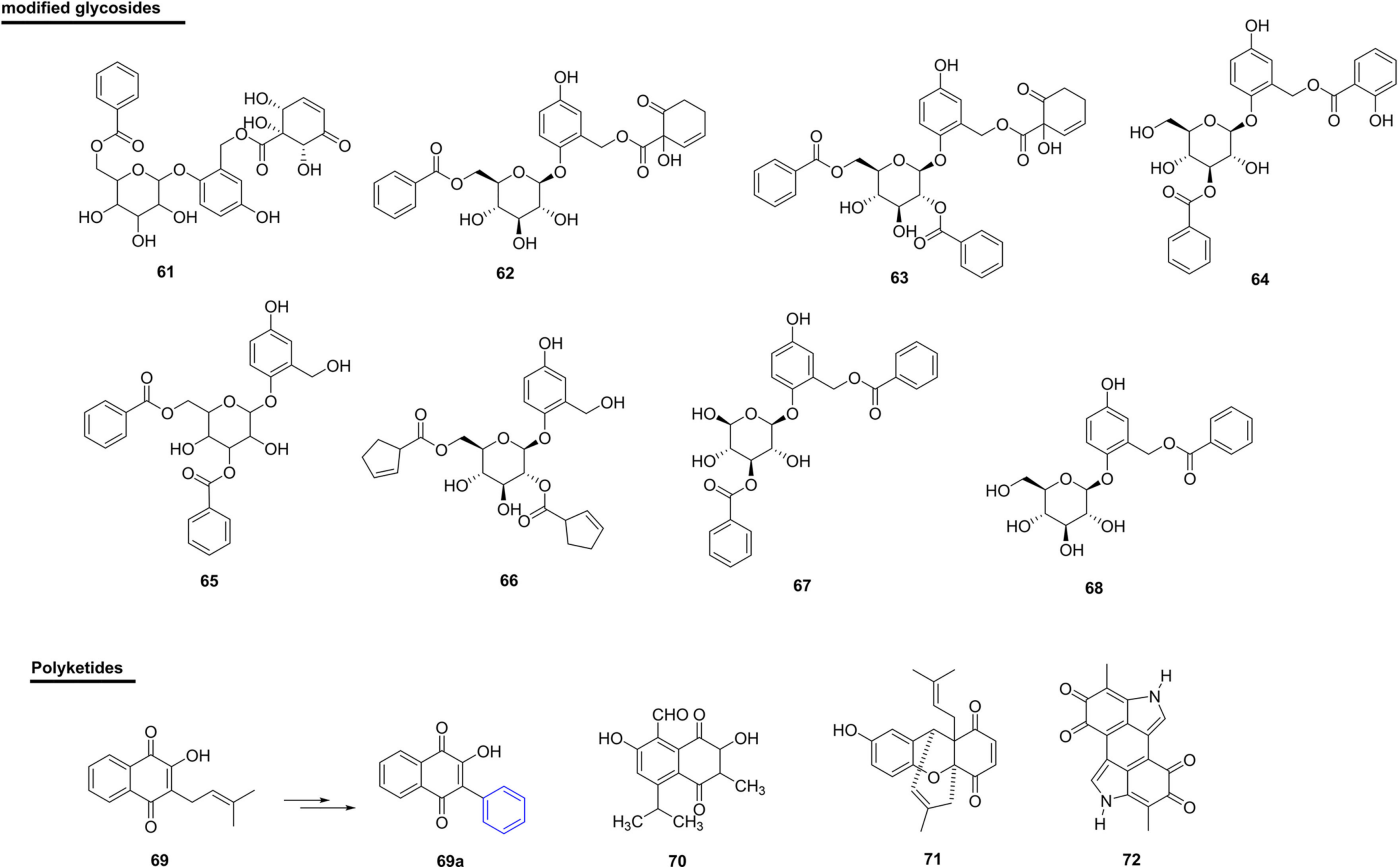
Chemical structures of snakebite treatment compounds **61-72**.

The compound 2-(6-benzoyl-*β*-glucopyranosyloxy)-7-(1*α*,2*α*,6*α*-trihydroxy-5-oxocyclohex-3-enoyl)-5-hydroxybenzyl alcohol (**61**), isolated from the bark and branches of *Bennettiodendron leprosipes* and *Flacourtia ramontchi* (Salicaceae) (used as a folk medicine), presented 14% inhibition against PDE-I (111). In this same study, the homaloside D (**62**) showed activity similar to 61, with 13% inhibition. Itoside B (**63**) and itoside F (**64**), isolated from *Itoa orientalis* (Salicaceae), showed 21% and 13% inhibition, respectively, against PDE-I (111) (**Figure 7** and **Table 4**). Despite being active, all modified glycosides showed that they possessed lower potential when compared with other natural product classes.

**Table 4:** Modified glycosides **(61-68)** and polyketides **(69-72)** with antivenom properties.

N°	Compound	Plant	Activity inhibited	Venom or toxin	Snake	Reference
**61**	2-(6-benzoyl-*β*-glucopyranosyloxy)-7-(1*α*,2*α*,6*α* -trihydroxy-5-oxocyclohex-3-enoyl)-5-hydroxybenzyl alcohol	*Bennettiodendron leprosipes*^B^*, Flacourtia ramontchi*^BC^	enzimatic	PDE-I	*-*	(111)
**62**	homaloside D	*B. leprosipes*^B^*, F. ramontchi*^BC^	enzimatic	PDE-I	*-*	(111)
**63**	itoside B	*Itoa orientalis*^B^	enzimatic	PDE-I	*-*	(111)
**64**	itoside F	*I. orientalis*^B,BC^	enzimatic	PDE-I	*-*	(111)
**65**	scolochinenoside C	*S. chinensis*^S^	enzimatic	PDE-I	*-*	(110)
**66**	scoloposide C	*S. chinensis*^S^	enzimatic	PDE-I	*-*	(110)
**67**	benzoylsalireposide	*Symplocos racemosa*^WP^	enzimatic	PDE-I	*-*	(147)
**68**	salireposide	*S. racemosa*^WP^	enzimatic	PDE-I	*-*	(147)
**69**	lapachol	*-*	enzimatic	Venom	*B. jararaca, B. atrox*	(180)
**69a**	analogue of lapachol	*-*	enzimatic	Venom	*B. jararaca, B. atrox*	(180)
**70**	isohemigossypolone	*Pachira aquatica*^R^	injury	Venom	*B. pauloensis, B. moojeni*	(149)
**71**	ehretianone	*Ehretia buxifolia*^SB^	morthality	Venom	*E. carinatus*	(86)
**72**	melanin	*Thea sinensis* Linn.^BT^	enzimatic	Venom, PLA_2_	*A. contortrix laticinctus, A. halys blomhoffii, C. atrox*	(150)

SB, stem bark; R, roots; S, stem; WP, whole plant; B, bark; BC, branches; BT, black tea; Phosphodiesterase I, PDE-I; Phospholipase A_2_, PLA_2_.

Two new phenolic glycosides, scoloquinenoside C (**65**) and scoloposide C (**66**), were isolated from the stem of *S. chinensis* (Salicaceae) in China. In addition, phenolic glycosides have shown inhibitory activity against PDE-I from snake venom (110).

Another study also showed inhibitory activity against PDE-I from snake venom, in which two new phenolic glycosides called benzoylsalreposide (**67**) (IC_50_ of 171 µM) and salireposide (**68**) (IC_50_ of 171 µM) were used. These were isolated from *Symplocos racemosa* (Symplocaceae) from Pakistan (147, 179) (**Figure 7** and **Table 4**).

### Polyketides

The natural naphthoquinone, lapachol (**69**), was isolated from the species *Tabebuia impetiginosa* (Bignoniaceae) from Brazil (state of Rio de Janeiro), and has been used as a starting point for obtaining new bioactive quinones (180). In this same work, an analogue of this natural compound (**69a**) showed the ability to antagonize the proteolytic activity (3-100 µM) and collagenase activity (10-100 µM) of *B. atrox* venom. In addition, *in vivo* pre-incubation of the venom with compound **69a** at concentrations of 1 mg/kg and 3 mg/kg eliminated the hemorrhage induced by the venom of *B. atrox* and, in relation to the venom of *B. jararaca*, the inhibition was greater than 70% with 10 mg/kg of the compound. The authors attributed the protective effect of the analogue of **69a** in the skin to the inhibition of proteolytic activities and collagenase, i.e., this compound may be interesting for preventing degradation of the vessels (180).

Another naphthoquinone, isohemigossypolone (**70**), isolated from the roots of *Pachira aquatica* (Malvaceae) from Brazil (state of Bahia), was able to significantly inhibit the *in vitro* coagulant activity of *B. pauloensis* venom. In *in vivo* experiments, the compound was able to significantly inhibit myotoxic activity caused by *B. pauloensis* venom, as well as neutralize the metalloproteinase activity of the whole venom by 70% and of the isolated SVMP (BthMP) by 40% (149) (**Figure 7** and **Table 4**).

Selvanayagam et al. isolated a quinonoid xanthene from the root bark of the species *Ehretia buxifolia* (Boraginaceae) from India (Tamilnadu), which is used as an antidote for *E. carinatus* envenomation (86). The compound ehretianone (**71**) was isolated from the methanolic extract of the bark of the plant species and was tested for antivenom activity against envenomations by *E. carinatus* in mice. In prophylactic treatment, the dosage of 3.75 mg/kg was administered 30 min before venom injection and mortality was reduced by 35% when compared to the controls. In the curative study, the same dosage of the compound gave significant protection up to 5 min after the injection of venom.

Melanin (**72**) extracted from black tea is a non-hydrolyzed complex of tea polyphenols and has been tested for its effect on venoms of the snakes *A. contortrix*, *A. halys* and *C. atrox*. In *in vitro* assays, there was a 43% decrease in the specific activity of the PLA_2_s enzyme and, in *in vivo* experiments, there was a significant increase in the survival time of mice after administration of the venoms of the three snake species (150).

### Terpene Compounds

**Figure 8:**
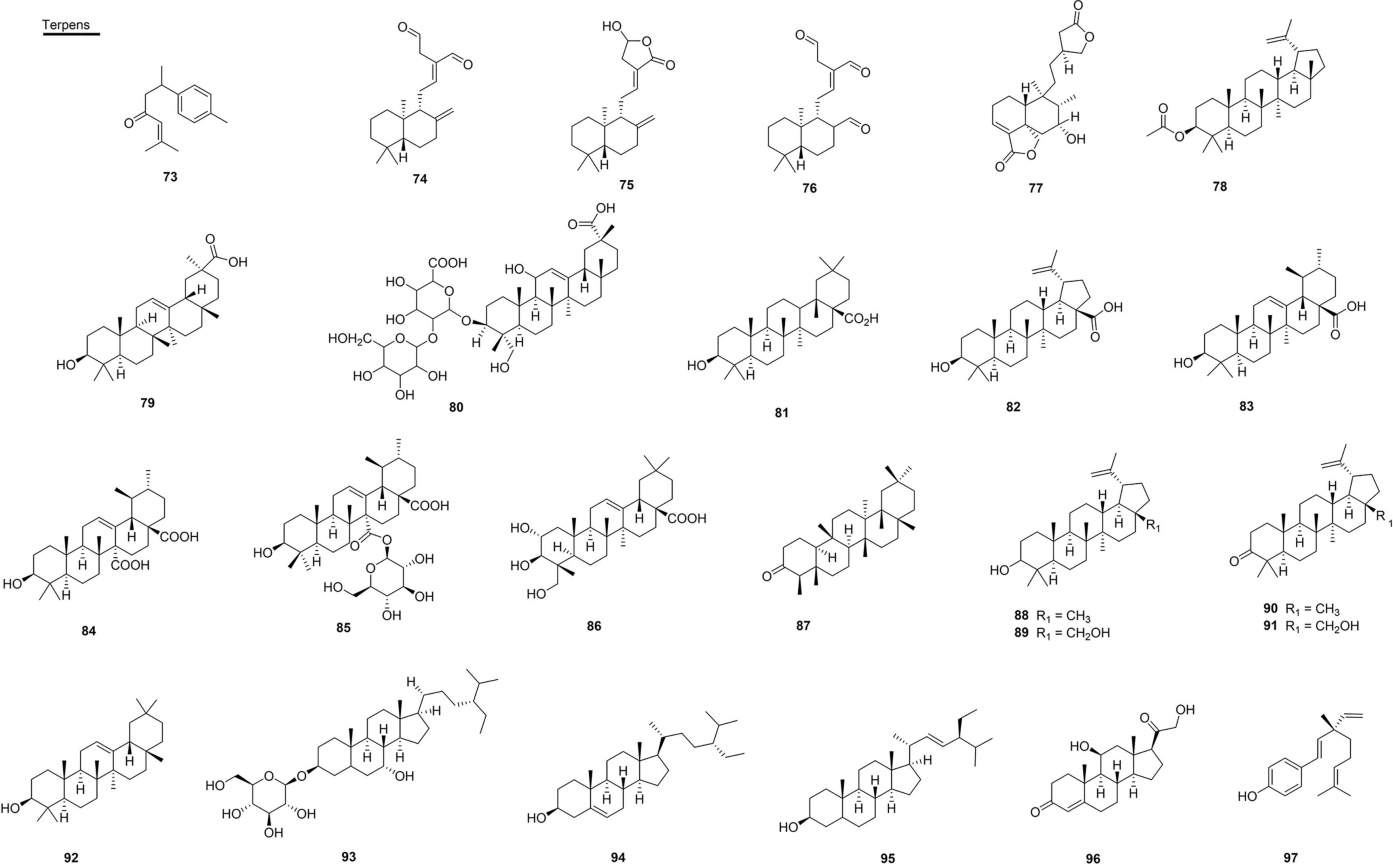
Chemical structures of snakebite treatment compounds **73-97**.

The sesquiterpene ar-turmerone (**73**), isolated from the roots of *Curcuma longa* (Zingiberaceae) from Brazil (state of Minas Gerais), was able to neutralize the hemorrhagic activity present in the venom of *B. jararaca* and reduced the lethal effect of the venom of *C. durissus* in rats by 70% (108). Another study performed with this compound and the venom of *B. alternatus* found that, in the first treatment, the hemorrhagic activity presented a reduction of the hemorrhagic halo of 3.82 mm by 0.31 mm, and also decreased edema. In addition, the necrosis that had occurred was reversed in all animals in a period of 96 h (137) (**Figure 8** and **Table 5**).

**Table 5:** Terpenes **(73-97)** with antivenom properties.

N°	Compound	Plant	Activity inhibited	Venom or toxin	Snake	Reference
**73**	ar-turmerone	*Curcuma longa*^R^	hemorrhagic, lethality, edematogenous, necrosis	Venom	*D. r. puchella, B. jararaca, C. d. terrificus*	(108, 137)
**74**	(*E*)-17-ethyliden-labd-12-ene-15,16-dial (labdane dialdehyde)	*C. zedoaroides*^RZ^	lethality	Venom	*B. asper* and *B. atrox*	(106)
**75**	labdane lactone	*C. antinaia*^RZ^*, C. contravenenum*^RZ^*, C. zedoaroides*^RZ^	lethality	Venom	*O. hannah*	(105)
**76**	labdane trialdehyde	*C. antinaia*^RZ^*, C. contravenenum*^RZ^*, C. zedoaroides*^RZ^	diaphragmatic, neurotoxic	Venom	*O. hannah*	(105)
**77**	neo-clerodane	*B. trimera*^A^	hemorrhagic	Venom, SVMP	*O. hannah*	(114)
**78**	lupeol acetate	*H. indicus*^R^	lethality, hemorrhagic, desfibrogenation, edematogenous, enzymatic, cardiotoxicity, neurotoxicity	Venom, PLA_2_	*B. neuwiedi, B. jararacussu*	(155)
**79**	11-deoxoglycyrrhetinic acid	*Clematis gouriana*^R^	enzimatic	PLA_2_	*D. russelii, N. kaouthia*	(135)
**80**	SID 249494135	*C. gouriana*^R^	enzimatic	PLA_2_	*D. russelii, N. kaouthia*	(135)
**81**	oleanolic acid	*B. uncinella*^A^	enzimatic, proteolytic, hemorrhagic, edematogenous	PLA_2_, SVMP	*D. russelii*, *N. naja, B. atrox, C. d.terrificus*	(112, 172, 181)
**82**	betulinic acid	*-*	proteolytic	Venom	*B. atrox*	(172)
**83**	ursolic acid	*B. uncinella*^A^	proteolytic, enzimatic, edematogenous	Venom, PLA_2_	*B. atrox, C. d. terrificus*	(112, 172)
**84**	quinovic acid	*Mitragyna stipulosa*^B^	enzimatic	PDE-I	*-*	(183)
**85**	quinovin glycoside C	*M. stipulosa*^B^	enzimatic	PDE-I	*-*	(183)
**86**	arjunolic acid	*Combretum leprosum*^R^	lethality, hemorrhagic, myotoxicity	Venom	*B. jararacussu, B. jararaca*	(139)
**87**	friedelin	*E. ovalifolium*^S^	hemorrhagic	Venom	*L. muta*	(168)
**88**	lupeol	*E. subsessile*^S^	proteolytic, hemolytic, hemorrhagic	Venom	*L. muta*	(168)
**89**	betulin	*Dipteryx alata*^B^	neuromuscular blocked	Venom	*B. jararacussu*	(97)
**90**	lupenone	*D. alata*^B^	neuromuscular blocked	Venom	*B. jararacussu*	(97)
**91**	28-OH-lupenona	*D. alata*^B^	neuromuscular blocked	Venom	*B. jararacussu*	(97)
**92**	*β*-amyrin	*Apuleia leiocarpa*	lethality	Venom	*B. jararaca*	(103)
**93**	ikshusterol 3-*O*-glucoside	*C. gouriana*^R^	enzimatic	Venom, PLA_2_	*N. naja*	(136)
**94**	*β*-sitosterol	*Pluchea indica*^R^	lethality, hemorrhagic, defibrogenation, cardiotoxicity, neurotoxicity, edematogenous, enzimatic	Venom, PLA_2_	*D. russelii, N. kaouthia*	(113)
**95**	stigmasterol	*P. indica*^R^	lethality, hemorrhagic, defibrogenation, cardiotoxicity, neurotoxicity, edematogenous, enzimatic	Venom, PLA_2_	*D. russelii, N. kaouthia*	(113)
**96**	corticosterone	*-*	enzimatic	PLA_2_	*D. russelii*	(156)
**97**	bakuchiol	*Psoralea corylifolia*	enzimatic, coagulant	Daboxin (PLA_2_)	*D. russelii*	(174)

R, roots; S, stem; SS, semi-synthetic; P, purchased (synthetic); A, aerial; B, bark; RZ, rhizomes. Phosphodiesterase I: PDE-I; Phospholipase A_2_: PLA_2_; Metalloproteinase: SVMP.

In relation to the diterpenes, (*E*)-17-ethyliden-labd-12-ene-15,16-dial (labdane dialdehyde) (**74**), isolated from the species *C. zedoaroides* (Zingiberaceae) from Thailand, showed an increase in the antagonistic antivenomous effects of (*O. hannah*) in a dose-dependent manner up to 32 µg/mL *in vitro*, and, when pre-incubated for 1 h, complete neutralization of the activity of the venom (from *O. hannah*) occurred (106). In addition, intraperitoneal injection of **74** at 100 mg/kg showed a significant effect of > 80% on the survival rate. Lattmann et al. (2010) showed that **74** was able to antagonize the action of snake venom at the neuromuscular junction, protecting mice from the lethal effects of raw venom (106). In this way, this compound acted as a mold for molecular recognition that was able to guide the dialdehyde irreversibly to the peptide target of the venom, and the complex that was formed was unable to block the nAChRs (**Figure 8** and **Table 5**).

In a subsequent study, Salama et al. isolated the diterpenes **74**, labdane lactone (**75**) and labdane trialdehyde (**76**) from species of the genus *Curcuma* (Zingiberaceae), also in Thailand (105). Compounds **75** and **76** (10 µg/mL), isolated from *C. antinaia* and *C.* zedoaroides, showed 83% and 62% inhibition, respectively, against *O. hannah* venom. Substance **76**, obtained from *C. contravenenum* extract, maintained diaphragmatic contraction almost in its entirety with 99% protection, and is thus considered the best antivenom and anti-neurotoxic agent, followed by **74** and **75** (105).

The compound *neo*-clerodane (**77**), isolated from the aerial parts of the plant *Baccharis trimera* (Asteraceae) from Brazil (state of São Paulo), significantly inhibited the hemorrhagic activity of venoms from *Bothrops* spp. and of the isolated SVMP Bjussu-MP-I from *B. jararacussu* venom (114). This compound was also able to inhibit the proteolytic activity of crude venom (70%) and classes of P-I and III SVMPs by over 80%, and also presented antimiotoxic and antiedematogenic properties induced by the venom of *B. jararacussu* (114) (**Figure 8** and **Table 5**).

Within the subclass of triterpenes, lupeol acetate (**78**) is isolated from the roots of *H. indicus* (Asclepiadaceae) and is found in several other plants that are studied regarding antivenom activity. This compound significantly neutralized lethality, hemorrhagic, defibrinogenation, edematogenic and PLA_2_s activities induced by *D. russelii* venom. It also neutralized lethality, cardiotoxicity, neurotoxicity and respiratory alterations caused by *N. kaouthia* venom. Compound **77** induced greater protection against the venoms of *D. russelii* and *N. kaouthia* when administered together with the antivenom compared to the action of treatment only with antivenom (155).

Two triterpenes were isolated from the roots of *Clematis gouriana* (Ranunculaceae) located in India, namely 11-deoxoglycyrrhetinic acid (**79**) and the new glycosidic terpene called SID 249494135 (**80**), both of which showed efficacy in inhibiting the enzyme PLA_2_s at the concentration of 1 µg/mL of the venom from *N. naja in vitro*. *In silico* assays showed that both compounds were well inserted in the active site of PLA_2_s (135).

Oleanolic acid (**81**) is the main metabolite found in several medicinal plants used in the treatment of inflammatory disorders. This metabolite was able to inhibit 20 µM PLA_2_s (> 90% and 83%) of the venom of *D. russeli* and *N. naja* with an IC_50_ of 3.08 and 7.78 µM, respectively (181). In addition, 81 inhibited indirect hemolytic activity and PLA_2_s-induced mouse paw edema *in vivo*. Further studies were conducted with the PLA_2_s of *D. russelii* venom and revealed that inhibition by **81** is not dependent on the substrate and calcium, and causes an inhibition that is irreversible. Results presented by Dharmappa et al. (181) also showed the inhibition of edema induction in a dependent concentration, in which, at 15 µM, edema rates decreased significantly, thus corroborating previous results and suggesting a strong correlation between the lipolytic and pro-inflammatory activity of **81** (181). This compound showed inhibition of proteolytic activity induced by the Batx-I SVMP from *B. atrox* venom, with an IC_50_ of 223.0 µM and hemorrhagic activity with an IC_50_ of 1 µM (172). In this same study, 81 displayed reductions in the formation of edema induced by metalloprotease at a dose-dependent concentration.

In addition, two other pentacyclic triterpenoids, betulinic acid (**82**) and ursolic acid (**83**) of synthetic origin, also inhibited the proteolytic activity of Batx-I with IC_50_ values of 115 and 357 µM, respectively. Additionally, inhibition of hemorrhagic activity was observed with an IC_50_ of 345 and 1.077 µM for **82** and **83** (172). Zalewski et al. (2011) evaluated the fraction composed of substances **82** and **83**, which were extracted from the aerial parts of the plant *Baccharis uncinella* (Asteraceae), and demonstrated strong potential for eliminating the PLA_2_s activity and PLA_2_s-induced edematogenic activity of *C. durissus* venom (112). Compound **83** was also effective in inhibiting the PLA_2_s enzyme of *D. russelii* venom and presented an IC_50_ of 12 µM and inhibition of hemolytic activity of 87%, besides being effective against *N. naja* venom with an IC_50_ of 18 µM and 73% in inhibiting the same activity for both at a concentration of 15 µM. It was also able to antagonize the induction of edema caused by *D. russelii* venom at a concentration of 12 µM (182) (**Figure 8** and **Table 5**).

The compounds quinovic acid (**84**) and quinovin glycoside C (**85**), isolated from the stem of the plant *Mitragyna stipulosa* (Rubiaceae), from Cameroon, showed significant inhibitory activity against PDE-I of snake venom with IC_50_ values of 0.166 and 0.374 mM, respectively (183).

Another compound was effective in neutralizing the venoms of *Bothrops* species; arjunolic acid (**86**), isolated from the roots of *Combretum leprosum* (Combretaceae) from Brazil (state of Ceará), was able to reduce lethality by more than 80% in the oral pretreatment performed, and it neutralized the myotoxic effect of the venom of *B. jararacussu*. Pre-incubation and pre-treatment with 30 mg/kg of 86 reduced the hemorrhagic activity of *B. jararaca* venom to 12% and 58%, respectively. In addition, at some concentrations, **86** inhibited some enzymatic activities, such as PLA_2_s, collagenase, proteolytic and hyaluronidase, of *B. jararacussu* and *B. jararaca* venoms at a dose-dependent concentration (139).

Isolated from the stem of the species of *E. ovalifolium* and *E. subsessile* (Erythroxylaceae) from Brazil (state of Rio de Janeiro), the compound friedelin (**87**) inhibited the hemorrhagic activity of the venom by 20%, and lupeol (**88**) inhibited the proteolytic and hemolytic activity of the venom of *L. muta* to 5% and was also able to inhibit the hemorrhagic activity by 28%, which are all modest activities (168).

The study by Ferraz et al. showed the efficacy of triterpenoids isolated from *D. alata* (Fabaceae) from Brazil (state of Tocantins), which were tested (0.2 mg/mL) against the irreversible neuromuscular blockade caused by *B. jararacussu* venom: **88** (70%), betuline (**89**) (68%), lupenone (**90**) (45%) and 28-OH-lupenone (**91**) (54%) (97). In addition, compounds 89 (39%) and **90** (49%) showed significant protection against *C. durissus* envenomations of the neuromuscular junction. In addition to these, the compound *β*-amyrin (**92**), isolated from *Apuleia leiocarpa* (Fabaceae) from Brazil (state of Rio de Janeiro), resulted in the survival of 60% of the animals that were tested 48 h after being given the venom of *B. jararaca* (103).

Within the subclass of the steroids, ikshusterol 3-*O*-glucoside (**93**), isolated from *C. gouriana* (Ranunculaceae) from India, showed a moderate inhibitory activity (30%) that was capable of neutralizing the action of *N. naja* venom at a concentration of 1,000 µg/mL. In addition, *in vitro* assays showed a good ability to inhibit the PLA_2_s (136). In *in vitro* studies, the mixtures of the compounds *β*-sitosterol (**94**) and stigmasterol (**95**), extracted from the roots (100 µg) of *Pluchea indica* (Asteraceae) in India, showed protection against lethality, hemorrhagic activity, defibrinogenation, cardiotoxicity, neurotoxicity, respiratory changes, and inhibition of the activity of PLA_2_s and the edema induced by the venom of *D. russelii* and *N. kaouthia* (113).

The synthetically obtained corticosteroid corticosterone (**96**) showed effective inhibitory activity of the enzyme PLA_2_s present in the venom of *D. russelii* with an IC_50_ of 30.4 µM (156). Bakuchiol (**97**), a synthetic meroterpenoid, inhibited the activity of the main PLA_2_s enzyme, daboxin P, with an IC_50_ of 744 µM (174) (**Figure 8** and **Table 5**).

### Saponins

**Figure 9:**
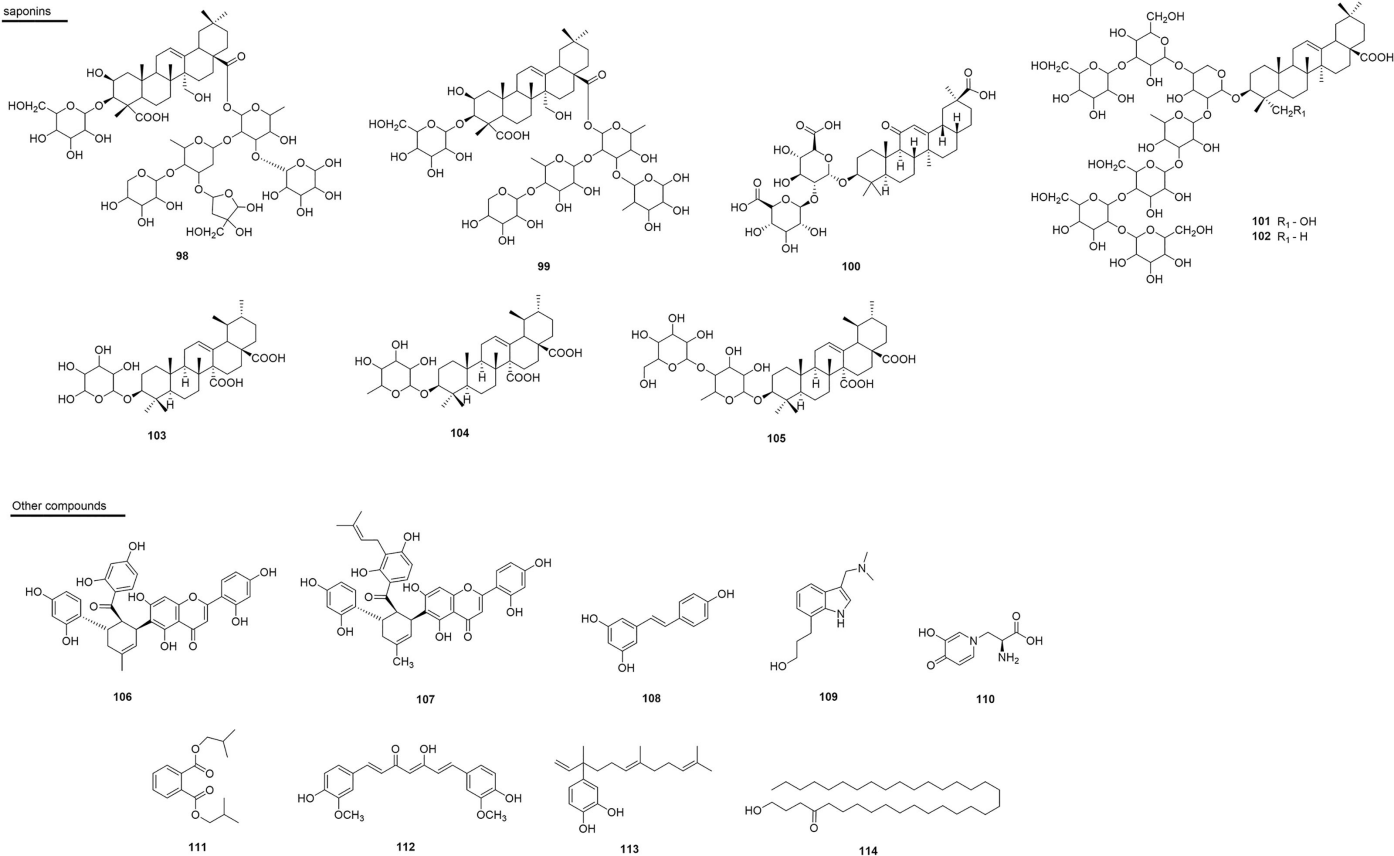
Chemical structures of snakebite treatment compounds **98-114**.

Some saponins have also been reported as snake venom inhibitors. The triterpene saponin bredemeyeroside B (**98**), isolated from the roots of *Bredemeyera floribunda* (Polygalaceae) from Brazil (state of Ceará), showed inhibitory action against the lethality of the venom of *B. jararaca* at a dose of 100 mg/kg (orally), resulting in the survival of 90% of the animals tested (148). From this same species, bredemeyroside D (**99**) was isolated, which was also able to show inhibitory action of the lethality of the venom of *B. jararaca* (100% of the mice after 6 h) under the same conditions carried out in the previous study (184). Glycyrrhizin (**100**), extracted from the roots of the plant *Glycyrrhiza glabra* (Fabaceae) from Brazil (state of São Paulo), had an *in vitro* inhibitory effect on human fibrogen coagulation induced by *B. jararaca* venom (IC_50_ 1.2 mM), hydrolytic activity *in vitro* (IC_50_ 0.47 mM) and inhibitory activity on platelet aggregation *in vitro* (IC_50_ 0.33 mM) (101). Additionally, in *in vivo* assays, compound **98** exhibited significant inhibition of 86% of thrombus weight against venom doses and eliminated venom-induced bleeding with co-administration of the substance together with antivenom (148) (**Figure 9** and **Table 6**).

**Table 6:** Saponins **(98-105)** and other compounds **(106-114)** with antivenom properties.

N°	Compound	Plant	Activity inhibited	Venom or toxin	Snake	Reference
**98**	bredemeyeroside B	*Bredemeyera floribunda*^R^	lethality	Venom	*B. jararaca*	(148)
**99**	bredemeyeroside D	*B. floribunda*^R^	lethality	Venom	*B. jararaca*	(184)
**100**	glycyrrhizin	*Glycyrrhiza glabra*^R^	coagulation, hidrolytic, platelet aggregation	Venom	*B. jararaca*	(101)
**101**	macrolobin A	*Pentaclethra macroloba*^B^	hemorrhagic, fibrogenolytic	Venom, SVMP	*B. neuwiedi, B. jararacussu*	(100)
**102**	macrolobin B	*P. macroloba*^B^	hemorrhagic, fibrogenolytic	Venom, SVMP	*B. neuwiedi, B. jararacussu*	(100)
**103**	quinovic acid-3-*O*-*α*-L-rhamnopyranoside	*Bridelia ndellensis*^B^	enzimatic	PDE-I	*-*	(185)
**104**	quinovic acid-3-*O*-*β*-D-fucopyranoside	*B. ndellensis*^B^	enzimatic	PDE-I	*-*	(185)
**105**	quinovic acid-3-*O*-*β*-D-glucopyranosyl (1 → 4)-*β*-D-fucopyranoside	*B. ndellensis*^B^	enzimatic	PDE-I	*-*	(185)
**106**	mesozygin B	*M. mesozygia*^L^	enzimatic	PDE-I	*-*	(186)
**107**	artonin I	*M. mesozygia*^L^	enzimatic	PDE-I	*-*	(186)
**108**	resveratrol	*-*	enzimatic	PLA_2_	*D. r. puchella*	(156)
**109**	gramine	*-*	enzimatic	PLA_2_	*D. r. puchella*	(156)
**110**	mimosine	*-*	enzimatic, myotoxicity	Venom, HAase	*D. russelii*	(174)
**111**	2-Methylpropyl phthalate	*Emblica officinalis*^R^	myotoxicity	Venom	*N. kaouthia, V. russelii*	(188)
**112**	curcumine	*-*	enzimatic	HAase	*N. naja*	(171)
**113**	4-nerolidylcatechol	*Piper umbellatum*^BC^*, P. peltatum*^BC^	enzimatic	PLA_2_, SVSP	*B. asper*	(189)
					*B. jararacussu*	
**114**	1-hydroxytetratriacontan-4-one	*Leucas aspera*^L^	venom action	Venom	*N. n. naja*	(138)

L, leaves; R, roots; B, bark; BC, branches. Hyaluronidase, HAase; Phosphodiesterase I, PDE-I; Phospholipase A_2_, PLA_2_; Metalloproteinase, SVMP; Serine protease, SVSP.

The compounds macrolobin A (**101**) and macrolobin B (**102**) were isolated from the bark of *Pentaclethra macroloba* (Fabaceae) from Brazil (state of Amapá), and significantly inhibited the hemorrhagic and the fibrogenolytic activities of *Bothrops* venoms and the SVMP Bjussu-MP-I from *B. jararacussu* venom, and were shown to be dose dependent (100). Compound **101** was more promising, with proteolytic activity of raw venom and fibrin SVMPs; classes I and III were inhibited by up to 90% and 80%, respectively. Regarding coagulation activity, **101** was able to completely inhibit *B. jararacussu* venom and the thrombin-like enzyme Bjussu-SP-I afterincubation periods of 1 h and 30 min, respectively (100).

The glycosidic derivatives of quinovic acid, first isolated from the bark of *Bridelia ndellensis* (Euphorbiaceae) collected in Cameroon (Ngaoundre), showed inhibitory activities against the enzyme PDE-I (phosphodiesterase-I). The compounds of quinovic acid-3-*O*-*α*-L-rhamnopyranoside (**103**), quinovic acid-3-*O*-*β*-D-fucopyranoside (**104**) and quinovic acid-3-*O*-*β*-D-glucopyranosyl (1 → 4)-*β*-D-fucopyranoside (**105**) were able to significantly inhibit the enzyme with an IC_50_ of 85 µM, 85 µM, and 75 µM, respectively (185) (**Figure 9** and **Table 6**).

### Other Compounds

Fozing et al. (186) demonstrated the inhibition of the enzyme phosphodiesterase I (of commercial origin) by compounds obtained from the leaves of *Morus mesozygia* (Moraceae). The compounds mesozygin B (**106**) and artonine I (**107**) showed the most potent activity, with an IC_50_ of 8.9 µM and 15.4 µM, respectively (186).

Other compounds have also shown effective inhibitory activity of enzymes present in snake venom. Stilbene resveratrol (**108**) and an aliphatic derivative polyamine gramine {3-[3-(dimethylaminomethyl)-1*H*-indol-7-YL] propan-1-ol} (**109**) were tested for the inhibition capacity of the PLA_2_s enzyme of *D. russelii* venom and presented an IC_50_ of 43.4 µM and 50.4 µm, respectively (156). In this same work, studies were carried out to verify the interactions of PLA_2_s with the compounds, in addition to determining the structures of PLA_2_s complexes with these compounds.

In *in vitro* experiments, the amino acid mimosine [*β*-3-(3-hydroxy-4-oxopyridyl) alpha-amino-propionic acid] (**110**) inhibited HAases (DRHyal-II) in a dose-dependent manner, and its activity with complete inhibition at 24 µM and an IC_50_ value of 12 µM. In addition, **110** also neutralized the same activity of *D. russelii* venom in a dose-dependent manner. The hyaluronidase activity of the venom was eliminated at 1000 µM with an IC_50_ value of 500 µM. In *in vivo* experiments, **110** inhibited DRHyal-II-potentiated myotoxicity of the myotoxin VRV-PL-VIII (myotoxic PLA_2_s) (187). In the study by Devi et al., this substance, which was obtained as a synthetic product, inhibited the *in vitro* activity of the PLA_2_s enzyme of the raw venom (5 µg/ml) of *N. naja* (47.0%), *E. carinatus* (47.0%) and *D. russelii* (27%) at a concentration of 1,200 µM. Additionally, compound **110** inhibited the PLA_2_s activity of daboxin P with an IC_50_ value of 674.3 µM, thus indicating its anticoagulant property (174).

Another study confirmed the phytomedicinal value of diisobutyl pthalate, 2-methylpropyl phthalate (**111**), present in the root of *Emblica officinalis* (Phyllanthaceae) from India, which antagonized the myotoxicity induced by the venom of *D. russelii*, shown by the decreased levels of the myotoxicity marker enzymes CPK and LDH (188). The inhibitory effect of the bioactive polyphenol curcumin (**112**) on the activity of the enzyme HAases purified from the venom of *N. naja* showed 91% inhibition of hyaluronidase (171).

In addition, plant extracts used in folk medicine were evaluated for inhibition of the enzymatic activity of myotoxin I and a PLA_2_s from *B. asper* venom. The compound 4-nerolidylcatechol (**113**), isolated from *Piper umbellatum* and *P. peltatum* (Piperaceae) from Costa Rica (Upala and Guapiles), inhibited the PLA_2_s activity of myotoxin I of *B. asper* and *B. atrox* at a time-and concentration-dependent dose (IC_50_ of 1 mM). This compound was also able to inhibit PLA_2_ss activity of group I of pseudexin and *Micrurus mipartitus* venom, and of group II as *Bothrops* toxins (189). Pre-incubation of **113** with the myotoxins of the two snakes showed a reduction in myotoxic activity by approximately 50% and the inflammatory response was significantly reduced in both (189). Additionally, when pre-treated with 0.4 mg of 113 up to 1 h prior to toxin administration, edema reduction by approximately one third was observed in *in vivo* studies. For Núñez et al., this compound also showed the ability to inhibit the proteolytic activity of trypsin on casein by 75%, in addition to eliminating in *in vitro* tests the pro-coagulant action of one SVSP that was isolated from the venom of *B. jararacussu* (189) (**Figure 9** and **Table 6**).

The fatty alcohol 1-hydroxytetratriacontan-4-one (**114**), isolated from the leaves of *Leucas aspera* (Lamiaceae), showed strong activity against the venom of *N. naja* in *in vivo* tests, with a mean effective dose (ED_50_) of 34.47 mg and full effective dose (ED_100_) of 68.93 mg per animal. A 100% survival rate was also proven when envenomed mice were treated at the same time with **114**, at the dosage of 75 mg per animal, in addition to significantly attenuating the antioxidant activity induced by the venom and the activity of lipid peroxidase in different organs (138).

### Proteins and Peptides

An acid glycoprotein, isolated from the species *Withania somnifera* (Solanaceae) from India and named WSG, has been identified as a possible inhibitor of snake venoms. In experiments with *N. naja* venom, the compound was able to inhibit PLA_2_s *in vitro* (ratio 1:2), and inhibited edema induction (concentration 1:2), and also neutralized the phospholipase-induced myotoxicity of Indian snake venom (*N. naja*) at the molar ratio of 1:2 (PLA_2_s:WSG) (153). In other studies, the same compound also inhibited the catalytic activity of different PLA_2_s isoforms of *N. naja* venom, increased the survival time of mice (152) and inhibited the activity of the HAases enzyme of the venoms of *N. naja* and *D. russelii* with an IC_50_ of 52.0 µg and 36.0 µg, respectively (151).

Turmerin, a protein of the Indian species *C. longa* (Zingiberaceae) (common name: turmeric) was effective in the inhibition of cytotoxicity in a dose-dependent manner, and also effectively inhibited the edema induced by phospholipase, which is a toxic venom of *N. naja* (PLA_2_s), in a molar ratio of 1:2.5 PLA_2_s:turmerin, also in a dose-dependent manner (107).

A study with different snake species showed that the protein concanavalin-A inhibited the 5’AMP enzyme of the species *N. naja, N. kauthia, N. melanoleuca*, *A. halys*, *B. asper*, *B. orientis* and *Oxyuranus scutellatus* with an IC_50_ of 0.2-1.2 µM (61).

## Perspectives for the Bioprospection of Antivenom Natural Products

Historically, natural products have played a key role in drug discovery and are invaluable resources that can contribute, especially as adjuvant inhibitors, to neutralizing the action of snake venom toxins. However, difficulties are still encountered with natural products in the process of developing new drugs, despite numerous examples of successful applications throughout history. These difficulties, which are centered on low yields, difficulties in purification, as well as high rates of rediscovery, are a recurring problem in research with natural products.

Recently, new tools for the study of natural products have been introduced, especially molecular biology techniques, which allow access to silenced or orphaned biosynthetic gene clusters; however, such applications have been mostly applied in microbial chemistry. Solutions for this type of study with plants have also been implemented, particularly based on transcriptomics (190). In addition, more sensitive analytical methods have been developed, which permit more comprehensive metabolomic analysis, which can be combined with new data analysis tools such as GNPS (Global Natural Products Social Molecular Networking) (190).

Although the vast majority of inhibitors of snake toxins originate in plants, given the lack of information on the natural products of these organisms against snake venom, microbial chemistry still represents a potential area to be explored. Another untapped potential target is marine organisms (also including microorganisms), which have already proven to be valuable sources of new lead compounds (191).

It is worth noting that natural products are still in second place, when compared to synthetic compounds, especially those that are easily obtained and have proven human safety *via* clinical trials. In particular, the repositioning of drugs emerges as a valuable strategy, especially nowadays due to the SARS-COV-2019 pandemic, which has forced us to search for new treatments. In the field of SBEs, much of the efforts focusing on the auxillary treatment relies on drugs of synthetic origin or those that have lower production costs. With this, the time interval between the identification of a potentially useful molecule and the approval for human use is lower due to the availability of safety data. Small molecules already provide an p-talternative application *via* the reuse of drugs in SBEs. Promising drug candidates such as batimastat, marimastat and varespladib have already advanced to phase II and phase III in preclinical trials (10, 192, 193). In this sense, several natural products and/or derivatives that have already been approved for human use, and/or molecules for which the safety is known, but somehow did not reach the final stages of the drug development, may be good candidates for future prospecting of toxin inhibitors that can serve as complementary treatments.

Combined with chemical methods, there is an increasing demand for methodologies that are capable of adequately simulating more complex biological conditions in order to better evaluate the effects of extracts and isolated natural products. Efforts such as the development of models with zebrafish, and other organisms, have met both the demands of researchers and the demands of more ethical laboratory practices. As such, knowledge regarding the chemical composition of the venom of the snake of origin is fundamental, since it allows the rationalization of studies aimed at the isolation of toxins. With the purified toxins, there is the possibility of their use in assays, as well as the information on crystalline structures, which aids studies on their mechanism of action, and also encourages computational research on structure-activity.

## Concluding Remarks

Discovering and developing molecules as new drug candidates is a complex long-term task with many obstacles, and this process involves a high cost. However, history has shown that research on natural products still has much to contribute to the advancement of knowledge for solving public health problems, especially those that are often neglected such as SBEs. Plants have served as important sources of medicine for snakebite complications, and this is attributed to the presence of several chemical compounds that are capable of inhibiting venom toxins. In this sense, this review sought to provide a more comprehensive knowledge of natural inhibitors isolated from plants for use against venoms and toxins and, in some way, contribute to the knowledge of potential options for auxillary treatment of SBEs. According to the papers analyzed, it was possible to identify 117 natural inhibitors from around the world that are commonly used as snake venom inhibitors. These findings deserve further attention and further studies in pre-clinical trials involving animals to direct future clinical applications in humans. It is important to use natural compounds as a combined therapy with the use of antivenoms to complement and/or improve serum therapy, thus enabling better neutralization of venom toxins and a reduction of human suffering caused by SBEs.

## Author Contributions

AA, AS, MS, WM, and HK conceived the main idea of this work. AA, AS, EL, WP, JM, and FS conducted the bibliography search. AA, AS, EL, WP, JM, and HK designed and wrote most of this review’s topics. AA, AS, and WP designed the figures of this review article. MP, AM-d-S, WM, MS, and HK corrected the manuscript and provided important contributions during the development of this work. All authors listed have made a substantial intellectual contribution to the work and approved it for publication.

## Funding

We would like to thank Fundação de Amparo à Pesquisa do Estado do Amazonas (FAPEAM) for the funding of HHFK’s research under the Universal Call FAPEAM-006/2019 and CT&I Priority Areas Call FAPEAM 010/2021 for HHFK and MS. HHFK and WMM acknowledges FAPEAM for funding *via* the calls PAPAC 005/2019, PRO-ESTADO and POSGRAD 2020-2021. The authors would also like to thank Conselho Nacional de Desenvolvimento Científico e Tecnológico (CNPq) for the payment of scholarships to MBP (No. 307184/2020-0), WM (No. 309207/2020-7), to AMMS (No. 303958/2018-9) and HK (No. 305942/2020-4). MP (Snakebite Roraima project coordinator) acknowledges funding support from the Hamish Ogston Foundation - Global Snakebite Initiative. This study was financed in part by the Coordenação de Aperfeiçoamento de Pessoal de Nível Superior – Brasil (CAPES) – Finance Code 001.

## Conflict of Interest

The authors declare that the research was conducted in the absence of any commercial or financial relationships that could be construed as a potential conflict of interest.

## Publisher’s Note

All claims expressed in this article are solely those of the authors and do not necessarily represent those of their affiliated organizations, or those of the publisher, the editors and the reviewers. Any product that may be evaluated in this article, or claim that may be made by its manufacturer, is not guaranteed or endorsed by the publisher.
